# Regulatory genome annotation of 33 insect species

**DOI:** 10.7554/eLife.96738

**Published:** 2024-10-11

**Authors:** Hasiba Asma, Ellen Tieke, Kevin D Deem, Jabale Rahmat, Tiffany Dong, Xinbo Huang, Yoshinori Tomoyasu, Marc S Halfon

**Affiliations:** 1 Program in Genetics, Genomics, and Bioinformatics, University at Buffalo-State University of New York Buffalo United States; 2 Department of Biology, Miami University Oxford United States; 3 Department of Biochemistry, University at Buffalo-State University of New York Buffalo United States; 4 Department of Biomedical Informatics, University at Buffalo-State University of New York Buffalo United States; 5 Department of Biological Sciences, University at Buffalo-State University of New York Buffalo United States; National Cancer Institute United States; National Cancer Institute United States

**Keywords:** insects, enhancers, regulatory genomics, genome annotation, enhancer prediction, *cis*-regulation, *D. melanogaster*, Other

## Abstract

Annotation of newly sequenced genomes frequently includes genes, but rarely covers important non-coding genomic features such as the *cis*-regulatory modules—e.g., enhancers and silencers—that regulate gene expression. Here, we begin to remedy this situation by developing a workflow for rapid initial annotation of insect regulatory sequences, and provide a searchable database resource with enhancer predictions for 33 genomes. Using our previously developed SCRMshaw computational enhancer prediction method, we predict over 2.8 million regulatory sequences along with the tissues where they are expected to be active, in a set of insect species ranging over 360 million years of evolution. Extensive analysis and validation of the data provides several lines of evidence suggesting that we achieve a high true-positive rate for enhancer prediction. One, we show that our predictions target specific loci, rather than random genomic locations. Two, we predict enhancers in orthologous loci across a diverged set of species to a significantly higher degree than random expectation would allow. Three, we demonstrate that our predictions are highly enriched for regions of accessible chromatin. Four, we achieve a validation rate in excess of 70% using in vivo reporter gene assays. As we continue to annotate both new tissues and new species, our regulatory annotation resource will provide a rich source of data for the research community and will have utility for both small-scale (single gene, single species) and large-scale (many genes, many species) studies of gene regulation. In particular, the ability to search for functionally related regulatory elements in orthologous loci should greatly facilitate studies of enhancer evolution even among distantly related species.

## Introduction

The past two decades have witnessed an explosive rise in sequenced metazoan genomes, from a mere handful in the first few years of the century to over 8000 today (NCBI, 2024; accessed January 16, 2024). This impressive statistic, however, masks the reality that these genomes exist in various stages of completion. Fewer than 30% of these genomes are assembled at the chromosome level, and only 28% of those have a comprehensive annotation (NCBI, 2024). Moreover, almost none of the genome annotations include regulatory sequences (also referred to as *cis*-regulatory modules [CRMs]) such as enhancers and silencers. This is unfortunate, as CRMs comprise a significant percentage of the genome, and knowledge of these sequences is expected to have value comparable to that of knowing the protein-coding genes. Characterizing CRMs is critical for understanding mechanisms of gene regulation and the organization of gene regulatory networks. Moreover, the role of regulatory mutations is increasingly recognized as a driver of both evolution and disease (Carroll et al., 2005; Carroll, 2008; Claringbould and Zaugg, 2021; Rickels and Shilatifard, 2018; Smith and Shilatifard, 2014).

One reason for the overall dearth of regulatory annotations is that, historically, large-scale CRM discovery has been difficult and both resource and labor intensive (Suryamohan and Halfon, 2015). For much of the last four decades, CRMs could only be identified through painstaking, low-throughput experimental assays. Although in recent years high-throughput empirical and computational CRM discovery methods have been developed, the various different methods frequently show limited agreement (Benton et al., 2019; Halfon, 2019; Lindhorst and Halfon, 2023), with the result that comprehensive CRM annotation across all cell types and life-cycle stages has remained a challenge for all but the most exhaustively studied model organisms. The problem is particularly acute for the insects. Insects represent a species-rich class—they constitute somewhere between 65% and 90% of all animal species (IUCN, 2022; Royal Entomological Society, 2023)—and have major impacts on human health and agriculture. The early radiation of the insects, coupled with typically short generation times, means that most of the relevant biomedically and agriculturally important species share little non-coding sequence conservation with each other or with the principal insect model species, *Drosophila melanogaster*. Thus, common sequence-homology-based CRM discovery approaches are of little use in providing regulatory insights into these species, and knowledge transfers poorly from one species to another. Furthermore, many insects have a complex and varied life cycle, making it particularly important—yet onerous—to assay for CRM function at multiple stages.

We previously developed a powerful computational method, SCRMshaw (‘Supervised *Cis*-Regulatory Module prediction’), for accurate prediction of CRMs, particularly enhancers (Kantorovitz et al., 2009; Kazemian et al., 2011; Kazemian and Halfon, 2019). (Although we expect SCRMshaw to be adept at finding multiple CRM types, our validation efforts to date have focused solely on enhancers.) SCRMshaw requires only a sequenced genome and a ‘training set’ of some 15–30 known enhancers that regulate a common pattern of gene expression (e.g. midgut expression) and relies on the idea that enhancers with similar function will have similar sequence characteristics, not possible to detect by eye or by traditional alignment methods, but identifiable using machine learning. Although not universally true, this assumption is robust enough to allow effective enhancer discovery without requiring knowledge of transcription factor binding sites or of the expression patterns of the genes being regulated. Importantly, we have shown that we can leverage the wealth of existing *D. melanogaster* enhancer data (Keränen et al., 2022) to train models for *cross-species* supervised enhancer discovery in diverged (160–345 million years [MY]) insect species—including flies, mosquitoes, beetles, bees, and wasps—despite a virtually complete lack of observable alignment at the non-coding sequence level (Kazemian et al., 2014; Lai et al., 2018; Schember and Halfon, 2021; Suryamohan et al., 2016).

Here, we use SCRMshaw to undertake an initial regulatory annotation of 33 individual insect genomes, using a collection of 48 training sets composed of experimentally validated *D. melanogaster* enhancers. These species are spread across five orders spanning over 360 MY of evolution, and represent roughly 10% of annotated insect species with scaffold-level or better assembly. Annotated predicted enhancers are provided in a searchable database that allows querying by species, tissue/cell type, or potential target gene. A series of simulations as well as in silico and in vivo validation experiments demonstrate the effectiveness of our approach and place an upper bound on false-positive prediction rates. Our results represent the first release of a rich insect regulatory genome annotation resource, which will continue to grow and annotate insect regulatory genomes in parallel with the sequencing of new insect genomes.

## Results

**Figure 1.**
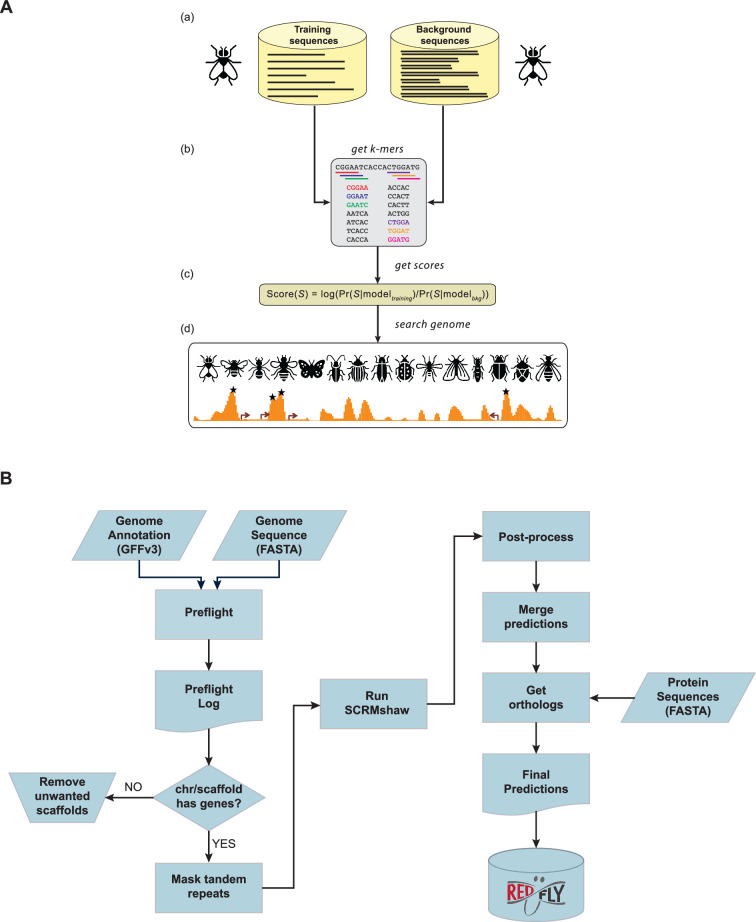
The SCRMshaw method and analysis pipeline. (**A**) Supervised motif-blind *cis*-regulatory module (CRM) discovery (SCRMshaw). (**a**) SCRMshaw uses a training set of known *D. melanogaster* enhancers (‘training sequences’), drawn from REDfly, that are defined by common functional characterization, and a 10‐fold larger background set of similarly sized common functional characterization, non‐enhancer sequences (‘background sequences’). (**b**) The short DNA subsequence (*kmer*) count distributions of these sequences are then used to train a statistical model. Note that although the pictured example shows 5-mers, *kmers* of different sizes are used for some of the underlying statistical models (see Methods). The trained model (**c**) is used to score overlapping windows in the ‘target genome’. (**d**) High-scoring regions are predicted to be functional regulatory sequences (asterisks). Figure adapted from Asma and Halfon, 2021. (**B**) The workflow used for the regulatory genome annotation described in this paper. The left side shows pre-processing steps, the right side, post-processing. Input to SCRMshaw consists of the genome sequence and gene annotation. A protein sequence annotation is supplied later for the orthology mapping step. Final results are made available as part of the REDfly regulatory annotation knowledgebase.

We previously demonstrated that SCRMshaw is remarkably effective at predicting enhancers across the entire ~345 MY range of the holometabolous insects, using training data derived solely from *D. melanogaster* (Kazemian et al., 2014; Schember and Halfon, 2021). SCRMshaw (Figure 1A) uses training sets composed of known enhancers defined by a common functional characterization (e.g. ‘nervous system,’ ‘wing disc’) to build a statistical model that captures their short DNA subsequence (*kmer*) count distribution. This *kmer* distribution is then compared to that of a set of non-enhancer ‘background’ sequences in a machine-learning framework. The *kmers* likely serve as proxies for the unknown transcription factor binding sites, but these sites themselves, even when known, are not explicitly used by the algorithm. The trained model is then used to score overlapping sequence windows in the genome, and the highest-scoring windows are output as predicted enhancers (Kantorovitz et al., 2009; Kazemian et al., 2011; Asma and Halfon, 2019). When searching the genomes of the mosquitoes *Anopheles gambiae* and *Aedes aegypti,* the red flour beetle *Tribolium castaneum,* the honey bee *Apis mellifera,* and the wasp *Nasonia vitripennis,* SCRMshaw successfully predicted enhancers in a cross-species fashion with an approximately 75% prediction success rate, based on reporter gene assays in xenotransgenic *D. melanogaster* (chosen as a pragmatic transgene host species) and comparison to already-identified enhancers in the other species (Kazemian et al., 2014; Lai et al., 2018; Schember and Halfon, 2021; Suryamohan et al., 2016). These results suggest that there are significant, albeit hidden, homologies governing the sequence characteristics of insect enhancers, at least for those involved in a substantial number of gene regulatory networks, and motivated us to apply SCRMshaw to a large and diverse set of sequenced insect genomes.

**Figure 1—figure supplement 1.**
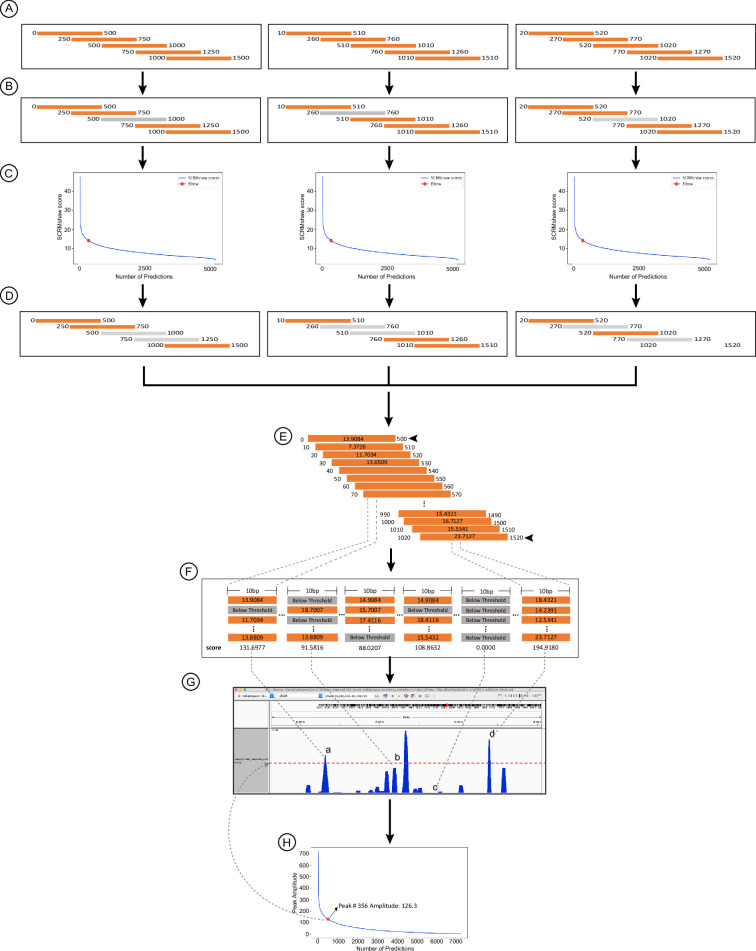
Revised post-processing method used for SCRMshaw. See Methods for details.

### A cross-species SCRMshaw pipeline

To facilitate application of SCRMshaw to large numbers of newly sequenced genomes, we developed a detailed workflow to ensure proper formatting of input genomes, rapid prediction of tissue-specific enhancer sequences, evaluation of results, and annotation of loci with information drawn from the respective orthologous regions in the richly investigated *D. melanogaster* genome (Figure 1B; see Methods). The workflow consists of four major steps:

#### Preflight

SCRMshaw requires two input files for each genome: a FASTA-formatted file of the genome sequence itself, and a GFFv3-formatted file of the genome annotation. The genome file is masked for tandem repeats using Tandem Repeat Finder (Benson, 1999). *Preflight* validates the formats of these files and produces a comprehensive log file that highlights any issues along with basic information such as the number of chromosomes/scaffolds and their sizes, data types present in the annotation (e.g. ‘gene’, ‘exon’, ‘ncRNA’, etc.), and average intergenic distances. *Preflight* also provides a sample output of the SCRMshaw-generated ‘gene’ and ‘exon’ files. This feature allows users to identify any discrepancies or errors stemming from the input files and to reformat these files as needed before running SCRMshaw. Any minor scaffolds that are annotated as not containing genes are discarded.

#### SCRMshaw

SCRMshaw is run as previously described (Kazemian and Halfon, 2019), using the ‘HD’ variant (Asma and Halfon, 2019). SCRMshaw_HD scans a genome with a 500 bp sliding window offset in 10 bp increments, using a set of three statistical models that compare the *kmer* composition of a set of training enhancers from *D. melanogaster* to randomly selected *D. melanogaster* non-coding sequences (see Methods).

#### Post-processing

The raw SCRMshaw output is post-processed to determine the final set of predicted enhancers. The original post-processing procedure described in Asma and Halfon, 2019, had a tendency to predict enhancers skewed toward long lengths (median ~1100 bp). We have revised that method here (see Methods) to yield predictions of more compact size (median 750 bp), which is more in keeping with empirically characterized enhancer lengths (Li et al., 2007).

#### Orthology mapping

Each SCRMshaw prediction is assigned its closest flanking genes as putative enhancer target genes, to aid in interpretation of the SCRMshaw output. There are clear shortcomings to this approach, as many enhancer targets are not the closest genes, leading to mis-assignments (e.g. Sanyal et al., 2012; Hafez et al., 2017; Chua et al., 2022; Qin et al., 2022). However, potentially more accurate methods for target-gene assignment rely on gene expression data, epigenetic data, and/or chromatin conformation data that are frequently not available for the species we are studying and thus difficult to incorporate into our prediction pipeline (Qin et al., 2022; Fishilevich et al., 2017; Gschwind et al., 2023; Whalen et al., 2016). Putative target genes are then mapped to their *D. melanogaster* orthologs (if an ortholog exists) using the *Orthologer* software from the Zdobnov lab (Kuznetsov et al., 2023) (see Methods). We use *D. melanogaster* as it has by far the most comprehensive gene annotation of the insects and thus provides the most detailed functional information for each gene. Mapping the genes from each species to a common ortholog helps us to assess whether we have obtained predicted enhancers in orthologous loci within the various species on which we have run SCRMshaw. The orthology mapping step requires that a set of predicted proteins is present as part of the existing genome annotation. Note that neither this step nor the earlier target-gene assignment step affects the enhancer predictions themselves, which are generated prior to target-gene assignment and orthology mapping and are considered equally valid regardless of putative target genes and whether or not orthologs can be matched to them.

### Annotation of 33 insect genomes

**Figure 2.**
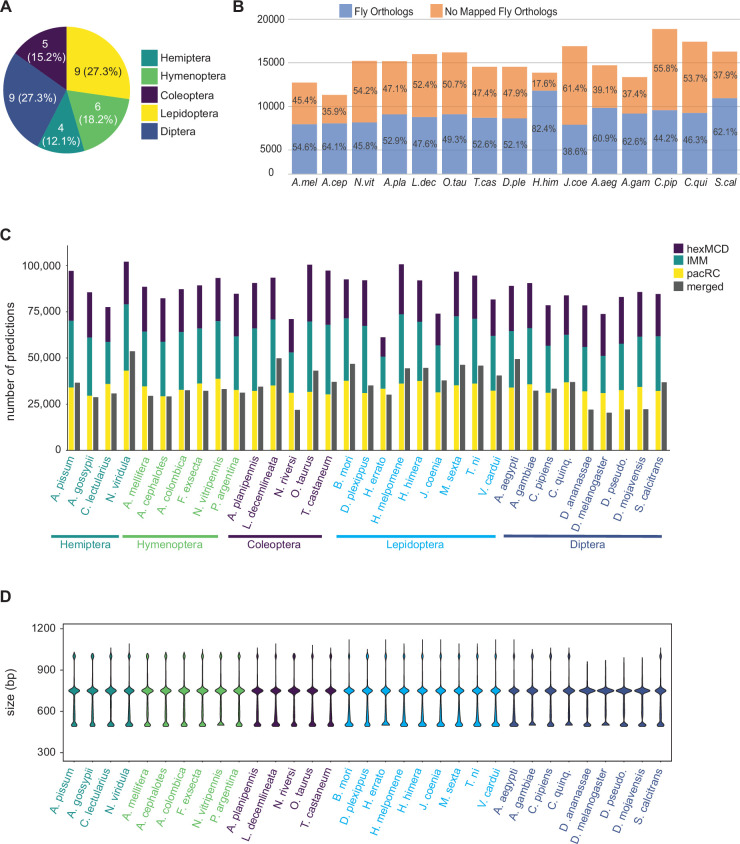
Annotation of 33 insect genomes. (**A**) Genomes from five insect orders were annotated in this study (more are ongoing). (**B**) Percentage of genes with *Drosophila* orthologs as mapped via our orthology pipeline (see Methods), for the 15 mapped species. ‘No Mapped Fly Orthologs’ indicates that our orthology mapping pipeline did not identify clear *D. melanogaster* orthologs. For any given gene, this could reflect either a true lack of a respective ortholog, or failure of our procedure to accurately identify an existing ortholog. For complete species names, see Table 1. (**C**) Total SCRMshaw predictions for each species. For each species, the left-hand column shows cumulative results for each SCRMshaw sub-method summed over each of the 48 training sets. The right-hand column shows the number of unique predictions after merging overlapping predictions from both sub-methods and training sets. Species are displayed alphabetically by taxonomic order (see also Figure 2—figure supplement 1). (**D**) Size distribution of SCRMshaw predictions, prior to merging overlapping predictions but after removing outlier predictions >2 kb in length. Species are ordered identically to panel C. Figure 2—source data 1.SCRMshaw training sets used in this study. Figure 2—source data 2.Number of predicted enhancers for each species, by method.

We ran our annotation pipeline on an initial set of 33 genomes (additional genome annotation is ongoing). These initial genomes were chosen based on availability and to sample broadly among the holometabolous insect orders and the Hemiptera (Figure 2A; Table 1). For each genome, we ran SCRMshaw using a collection of 48 training sets (Figure 2—source data 1) and all three SCRMshaw scoring methods (‘IMM’, ‘hexMCD‘, ‘PAC-rc’; Kantorovitz et al., 2009; Kazemian et al., 2011). For 15 species where a protein annotation was available, we assigned *D. melanogaster* orthologs to each predicted locus, with an average of 54% (range 38–82%) of genes in a given species having a *D. melanogaster* ortholog (Figure 2B). The complete data are available in multiple formats (see Data Availability).

**Figure 2—figure supplement 1.**
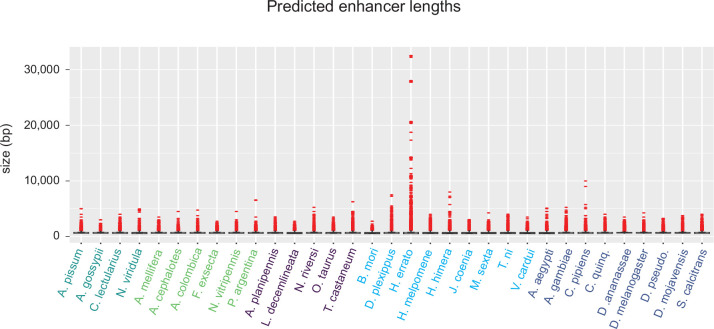
Lengths of predicted enhancers, including long outliers. Size distribution of SCRMshaw predictions, prior to merging overlapping predictions but without removing outlier predictions. Species are ordered as in Figure 2.

**Table 1.** Species used in this study.

Scientific name	Common name	Order	Assembly version/ annotation version	Link/URL for assembly information
*Acyrthosiphon pisum*	Pea aphid	Hemiptera	racon and v3_wdel	Courtesy Jennifer Brisson (University of Rochester)
*Aedes aegypti*	Yellow fever mosquito	Diptera	L5.2	https://www.ncbi.nlm.nih.gov/datasets/taxonomy/7159/
*Agrilus planipennis*	Emerald ash borer	Coleoptera	Apla_2.0	https://www.ncbi.nlm.nih.gov/datasets/taxonomy/224129/
*Anopheles gambiae*	African malaria mosquito	Diptera	P4.9	https://www.ncbi.nlm.nih.gov/datasets/taxonomy/7165/
*Aphis gossypii*	Cotton aphid/melon aphid	Hemiptera	ASM401081v2	https://www.ncbi.nlm.nih.gov/datasets/taxonomy/80765/
*Apis mellifera*	Honey bee	Hymenoptera	HAv3.1	https://www.ncbi.nlm.nih.gov/datasets/taxonomy/7460/
*Atta cephalotes*	Leafcutter ant	Hymenoptera	A.ceph_1.0	https://www.ncbi.nlm.nih.gov/datasets/taxonomy/12957/
*Atta colombica*	Leafcutter ant	Hymenoptera	Acol1.0	https://www.ncbi.nlm.nih.gov/datasets/taxonomy/520822/
*Bombyx mori*	Silkworm	Lepidoptera	ASM15162v1	http://ensembl.lepbase.org/Bombyx_mori_asm15162v1/Info/Index
*Cimex lectularius*	Bed bug	Hemiptera	Clec_2.1	https://www.ncbi.nlm.nih.gov/datasets/taxonomy/79782/
*Culex pipiens pallens*	Northern house mosquito	Diptera	TS_Cpip_V1	https://www.ncbi.nlm.nih.gov/datasets/taxonomy/42434/
*Culex quinquefasciatus*	Southern house mosquito	Diptera	VIPSU_Cqui_1.0_pri_paternal	https://doi.org/10.1093/gbe/evab005
*Danaus plexippus*	Monarch butterfly	Lepidoptera	v3	http://ensembl.lepbase.org/Danaus_plexippus_v3/Info/Index
*Drosophila ananassae*	Fruit fly	Diptera	caf1	http://ftp.flybase.net/genomes/Drosophila_ananassae/
*Drosophila melanogaster*	Fruit fly	Diptera	r6 1.8	https://www.ncbi.nlm.nih.gov/datasets/taxonomy/7227/
*Drosophila mojavensis*	Fruit fly	Diptera	caf1	https://www.ncbi.nlm.nih.gov/assembly/GCF_000005175.2/
*Drosophila pseudoobscura*	Fruit fly	Diptera	r3	http://ftp.flybase.net/genomes/Drosophila_pseudoobscura/dpse_r3.03_FB2015_01/
*Formica exsecta*	Wood ant	Hymenoptera	ASM365146v1	https://www.ncbi.nlm.nih.gov/datasets/genome/GCF_003651465.1/
*Heliconius erato*	Red postman butterfly	Lepidoptera	v1	http://ensembl.lepbase.org/Heliconius_erato_lativitta_v1/Info/Index
*Heliconius melpomene*	Postman butterfly	Lepidoptera	Hmel2	http://ensembl.lepbase.org/Heliconius_melpomene_melpomene_hmel2/Info/Index
*Heliconius himera*	False postman butterfly	Lepidoptera	Hed.V1	Courtesy Robert Reed (Cornell University)
*Junonia coenia*	Common buckeye butterfly	Lepidoptera	JC v1.0	Courtesy Robert Reed (Cornell University)
*Leptinotarsa decemlineata*	Colorado potato beetle	Coleoptera	Ldec_2.0	https://www.ncbi.nlm.nih.gov/datasets/taxonomy/7539/
*Manduca sexta*	Tobacco hornworm	Lepidoptera	v1.0	http://ensembl.lepbase.org/Manduca_sexta_msex1/Info/Index
*Nezara viridula*	Southern green stink bug	Hemiptera		https://www.ncbi.nlm.nih.gov/datasets/taxonomy/85310/
*Nasonia vitripennis*	Jewel wasp	Hymenoptera	Psr_1.1	https://www.ncbi.nlm.nih.gov/datasets/taxonomy/7425/
*Onthophagus taurus*	Dung beetle	Coleoptera	Otau_2.0	https://www.ncbi.nlm.nih.gov/datasets/taxonomy/166361/
*Pseudoatta argentina*	Leafcutter ant	Hymenoptera	ASM1760752v1	https://www.ncbi.nlm.nih.gov/datasets/taxonomy/621737/
*Stomoxys calcitrans*	Stable fly	Diptera	Stomoxys_calcitrans-1.0.1	https://doi.org/10.1186/s12915-021-00975-9
*Tribolium castaneum*	Red flour beetle	Coleoptera	r5.2	https://www.ncbi.nlm.nih.gov/datasets/taxonomy/7070/
*Trichoplusia ni*	Cabbage looper	Lepidoptera	tn1	https://www.ncbi.nlm.nih.gov/datasets/taxonomy/7111/
*Vanessa cardui*	Painted lady butterfly	Lepidoptera	Vcar_v1	Courtesy Robert Reed (Cornell University)
*Nebria riversi*	Ground beetle	Coleoptera	v1	https://doi.org/10.1111/1755-0998.13409

Collectively, we predicted a total of 2,873,192 enhancers in these 33 species, with each species having on average approximately 87,000 predictions (Figure 2—source data 2; Figure 2C). (As some enhancers are predicted by multiple scoring methods, or from more than one training set, the number of unique sequences is lower at 1,164,354; see gray bars in Figure 2C and Figure 2—source data 2.) The median length of the predicted enhancers across all species was 750 bp (mean 695, range 490–32,500 bp). However, we noted the presence of a small number—2642, <0.1% of the total—of unusually large regions (>2000 bp), the bulk of which were confined to just a few genomes (Figure 2—figure supplement 1). We therefore discarded any predictions with length greater than 1.5 times the interquartile range of the complete prediction set and re-evaluated the size distribution. This resulted in a median size of 740 bp with a mean of 676 bp and a range of 490–1120 bp, indicating that the overall impact of these outlier sequences is minimal (Figure 2D). Inspection of the excessively large elements revealed that they result from SCRMshaw predictions that lie immediately adjacent to each other (without gaps) and have similar SCRMshaw scores, which thus become merged into one broad predicted element. Preliminary analysis suggests that these regions result from insufficient masking of tandem repeat regions and/or genome assembly errors, although other causes, such as extremely enhancer-dense ‘superenhancer’ regions (reviewed by Grosveld et al., 2021), cannot entirely be ruled out.

### Many loci contain multiple enhancer predictions

We have noted in the past that SCRMshaw often predicts multiple enhancers in a single locus (e.g. Weinstein et al., 2023). This is consistent with the concept of ‘shadow enhancers’, sets of multiple redundant or semi-redundant enhancers regulating the same gene (reviewed by Kvon et al., 2021). On the other hand, if SCRMshaw is predicting enhancers with low specificity (i.e. largely at random), it is instead possible that larger loci may just accumulate a high number of predictions simply due to their greater length.

To distinguish between these possibilities, we conducted simulations on three representative genomes (*D. melanogaster, C. pipiens,* and *A. aegypti*), each with a different average intergenic region size chosen to represent small, medium, and large genomes respectively (average sizes 610 bp, 5274.5 bp, and 14,641 bp; see Methods). We randomized the location of SCRMshaw predictions across the non-coding component of each genome and compared the randomized results to our SCRMshaw output. We found that, for a subset of loci, the number of real SCRMshaw predictions per locus was consistently higher than the number obtained at random (Figure 3—source data 1). For example, using the *mapping1.visceral_mesoderm* training set, 4.6% (15/328) of *D. melanogaster* loci containing one or more SCRMshaw predictions had a significantly (p<0.001) larger number of predictions/locus than expected, 1.5% (10/658) of *C. pipiens* loci had more predictions than expected, and 1% (3/299) of *A. aegypti* loci had excess predicted enhancers (Figure 3—source data 1). Similarly, for the *mapping2.ectoderm* training set, 7.6%, 1.5%, and 11% of loci in the three species, respectively, had a significantly (p<0.001) greater than expected number of predictions per locus (Figure 3—source data 1). Averaged over all training sets, 3–4% of loci with predictions had significantly more than expected by chance (mean values: 3.0% for *D. melanogaster*, 3.0% for *C. pipiens*, and 3.7% for *A. aegypti*; maximum values: 10.2% for *D. melanogaster,* 9.3% for *C. pipiens*, and 11.6% for *A. aegypti*). Only very few training sets did not have any loci with significantly more enhancers than expected (1 set in *A. aegypti,* 1 in *C. pipiens,* and 8 in *D. melanogaster*; Figure 3—source data 1).

**Figure 3.**
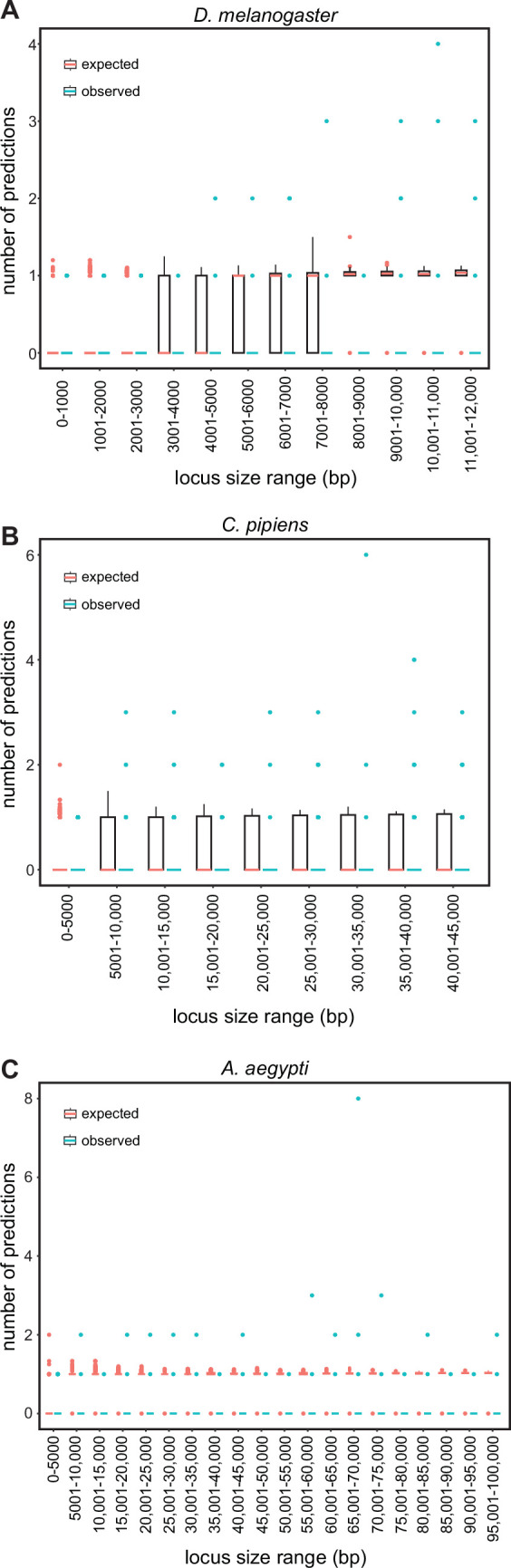
SCRMshaw makes multiple predictions per locus. The number of SCRMshaw predictions per locus (y-axis) are shown as boxplots for loci falling within the given size ranges (x-axis). Black boxes cover the 25–75th percentiles, bars indicate median values and dots indicate values exceeding 1.5 times the interquartile range (boxes are not visible for all bins due to very low degrees of variation). Values in pink represent expected values drawn from randomization, while values in blue represent observed values from SCRMshaw. All values are from results with the training set ‘mapping1.visceral_mesoderm’; results from other training sets were similar (see Figure 3—source data 1). Shown are results from the genomes of (**A**) *D. melanogaster*, (**B**) *C. pipiens*, and (**C**) *A. aegypti* representing small, medium, and large genomes, respectively. Figure 3—source data 1.Real and simulated predictions per locus.

To further ensure that these results were not influenced by locus size, we binned the loci by length and assessed the numbers of real versus simulated predictions/locus for each bin. The binned results were similar to the results using all loci, i.e., the number of SCRMshaw predictions/locus (Figure 3, blue boxplots) was consistently higher than the number obtained via randomization (Figure 3, pink boxplots). Results were also similar when comparing predictions only at intergenic versus only at intronic positions (Figure 3—source data 1). These results suggest that SCRMshaw is not predicting enhancers randomly throughout the genome, but rather is identifying multiple related ‘shadow’ enhancers in a subset of loci. Consistent with this interpretation, functional tests of the full set of predicted enhancers in several *D. melanogaster* loci has confirmed that many of the predictions act as functionally similar enhancers (T Williams, personal communication, December 2023).

### SCRMshaw predicts enhancers in orthologous loci across species

A major premise underlying cross-species applications of SCRMshaw is that conserved regulatory strategies should allow for a model trained on enhancers in one species to predict for similar enhancers in another species. We reasoned that at least some of the time, this should lead to identification of enhancers in orthologous loci, as orthologous genes are frequently involved in similar biological pathways and developmental regulatory networks (Carroll, 2008). Indeed, we previously showed that SCRMshaw was able to predict enhancers in several orthologous loci, for instance those for the *single-minded* locus in *D. melanogaster, A. gambiae,* and *A. mellifera,* and the *wingless* locus in *D. melanogaster, A. mellifera,* and *T. castaneum* (Kazemian et al., 2014). To test whether this is generally true, we used the 15 species with mapped *D. melanogaster* orthologs (plus *D. melanogaster* itself), and all 48 of our training sets, to compare the number of SCRMshaw-based versus random predictions obtained in common (orthologous) loci.

**Figure 4.**
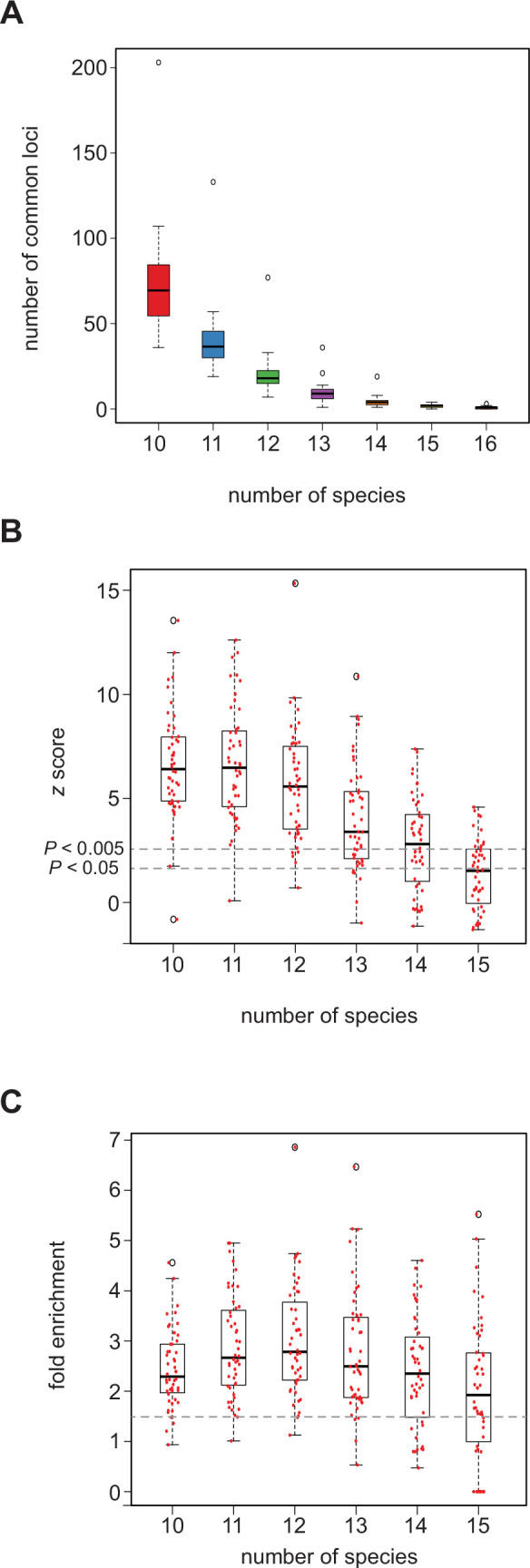
SCRMshaw predicts *cis*-regulatory modules (CRMs) in orthologous loci across species. (**A**) The number of loci in common that contain at least one SCRMshaw prediction, for 10 or more species. (**B**) z-scores demonstrating that the number of loci in common with one or more SCRMshaw predictions is significantly higher than expectation, based on 360 randomizations. The small number of common predictions for 14–16 species make these statistics unreliable. Dotted lines indicate z-score values representing significance at the (unadjusted) p<0.005 and p<0.05 levels. (**C**) Fold enrichment values illustrating the excess of loci in common with one or more SCRMshaw predictions compared to expectation. Dotted line shows 1.5× enrichment. Figure 4—source data 1.Real and simulated counts of predictions in orthologous loci.

We observed a substantial reduction in the number of orthologous loci with predictions in common as we moved from considering a set of 10 to the full set of all 16 species (average number of common loci over all 48 training sets: 70.7 (10 species)>39.4>19.8>9.14>4.12>1.6>0.6 (16 species) out of an average number of 640 predictions with mapped orthologs) (Figure 4A, Figure 4—source data 1, tab ‘a’). This rapid decline is likely due to a variety of factors, including differences in taxonomic order (e.g. Diptera vs. Hymenoptera), the quality and degree of ortholog data for each species, and the quality of annotation for each species, all of which likely lead to an underestimation of the true numbers of common loci in our data (see Discussion). We simulated SCRMshaw predictions for all 16 species by randomizing the SCRMshaw results and compared the number of common loci between the simulated and real data for combinations of 10–16 species. The number of common loci among the real SCRMshaw predictions was consistently significantly higher than the number of common loci observed in the simulated data (p<0.05; Figure 4B and Figure 4—source data 1). In particular, when evaluating between 10 and 12 species (data for 14–16 species are not reliable due to the very small number of observed loci in common) only a single training set, *adult_PNS*, did not have a significant overrepresentation of common loci (Figure 4—source data 1, tab ‘c’). We also examined the fold enrichment, i.e., the extent to which the true number was in excess of the simulated number, and found that on average there were greater than 2.4× more predictions in common loci than expected, when considering groups of 10–12 species; almost all training sets had a fold enrichment >1.5× (Figure 4C, Figure 4—source data 1, tab ‘d’). These results strongly suggest that SCRMshaw is successfully finding sets of conserved (or convergent) enhancers, as it is predicting sequences in orthologous loci in a training-set-specific manner across multiple species significantly more often than can be accounted for by chance.

### SCRMshaw predictions correlate with regions of accessible chromatin

Active enhancers are frequently found in regions of accessible chromatin, as assayed by methods such as DNAse-seq, FAIRE-seq (Formaldehyde-Assisted Isolation of Regulatory Elements with sequencing), and ATAC-seq (Assay for Transposase-Accessible Chromatin by sequencing) (Boyle et al., 2008; Buenrostro et al., 2013; Giresi et al., 2007). We confirmed previously that FAIRE-predicted and SCRMshaw-predicted enhancers in *T. castaneum* have a high (>79%) degree of overlap (Lai et al., 2018). To determine whether a similar correlation exists for other species, we compared our SCRMshaw predictions with available FAIRE-seq and ATAC-seq data for seven species: *D. melanogaster, T. castaneum, A. gambiae, Danaus plexippus, Vanessa cardui, Junonia coenia,* and *Heliconius himera*. These comparisons are imperfect, as the tissues used to obtain the chromatin data do not precisely correspond to the training sequences used for SCRMshaw, and the data were obtained using a variety of methods. Nevertheless, in the majority of cases where we were able to establish a rough match between the tissues, we observed significant overlap between the two methods of enhancer detection. For example, in *D. melanogaster*, 40% of SCRMshaw predictions using the *blastoderm.mapping1* training set overlapped ATAC-seq peaks obtained from blastoderm embryos (p<4.15e-112, fold enrichment 2.98) (Bozek et al., 2019; Table 2, row 2). Similarly, 35% and 41% of SCRMshaw predictions from the *mappng2.wing* and *haltere_disc* sets overlapped FAIRE data that included wing and haltere cells (McKay and Lieb, 2013) (p<4.6e-117 and 1.5e-102, fold enrichment of 3.29 and 2.98) (Table 2, rows 1, 3). In the mosquito *A. gambiae*, we compared SCRMshaw predictions from the *embryonic_midgut* and *mapping1.salivary* training sets to ATAC-seq data from adult midgut and salivary tissues, observing overlaps of 40% and 37% respectively (p<1.5e-102 and 2.15e-93, fold enrichment of 3.45 and 3.22) (Table 2, rows 9, 10). When comparing our SCRMshaw predictions from the *mapping2.wing* set to ATAC-seq data for larval wing tissue in the butterflies *D. plexippus* and *H. himera*, we observed overlaps of 60% and 68% (p<5.59e-19 and p<9.92e-25, fold enrichment of 1.99, 1.66) (Table 2, rows 11, 12) (Mazo-Vargas et al., 2022). The butterflies *J. coenia* and *V. cardui* are exceptions; intriguingly, they show a depletion in SCRMshaw predictions compared to expectation (Table 2, rows 13, 14). Whether this is due to a mismatch in the data used for comparison, the state of the genome assemblies for these two draft genomes, or some other failure of SCRMshaw to perform strongly on these species remains to be determined. Overall, the highly significant overlap we observe between SCRMshaw predictions and open chromatin regions in most species and tissues provides further strong evidence that SCRMshaw is effectively predicting enhancers across a broad range of genomes.

**Table 2.** Overlap of SCRMshaw predictions with FAIRE-seq and ATAC-seq peaks.

Species	Training set	Profiled tissue	Overlap(real)	Overlap (random)	s.d.*	z-score	FE^†^
*D. melanogaster*	haltere_disc	Wing, leg, and haltere third instar discs and pharate appendages; eye-antennal disc; third instar CNS	41.82%	14.02%	10.65	21.53	2.98
*D. melanogaster*	blastoderm.mapping1	Blastoderm	40.99%	13.86%	12.35	22.56	2.96
*D. melanogaster*	mapping2.wing	Wing, leg, and haltere third instar discs and pharate appendages; eye-antennal disc; third instar CNS	35.14%	10.69%	10.19	23.01	3.29
*T. castaneum*	mapping2.wing	Embryo, larval thoracic epidermis, larval brain	85.58%	26.18%	10.98	28.88	3.27
*T. castaneum*	mapping2.ectoderm	Embryo, larval thoracic epidermis, larval brain	81.68%	25.48%	12.48	26.29	3.21
*T. castaneum*	mapping1.ventral_ectoderm	Embryo, larval thoracic epidermis, larval brain	81.17%	24.76%	12.56	33.87	3.28
*T. castaneum*	mapping1.dorsal_ectoderm	Embryo, larval thoracic epidermis, larval brain	71.15%	24.69%	12.64	24.07	2.88
*T. castaneum*	mapping1.ectoderm	Embryo, larval thoracic epidermis, larval brain	69.53%	24.66%	11.92	22.35	2.82
*A. gambiae*	embryonic_midgut	Adult midgut, salivary_gland	40.20%	11.66%	7.90	21.56	3.45
*A. gambiae*	mapping1.salivary_gland	Adult midgut, salivary_gland	36.76%	11.43%	8.52	20.55	3.22
*D. plexippus*	mapping2.wing	Larval forewing, hindwing, head	60.43%	30.35%	6.30	8.93	1.99
*H. himera*	mapping2.wing	Larval forewing, hindwing, head	68.34%	41.10%	8.97	10.27	1.66
*J. coenia*	mapping2.wing	Larval forewing, hindwing, head	3.06%	34.91%	8.52	–12.22	0.09
*V. cardui*	mapping2.wing	Larval forewing, hindwing, head	15.46%	36.01%	8.01	–7.13	0.43

* Standard deviation.

† FE, fold enrichment.

### Reporter gene analysis demonstrates that SCRMshaw predictions are functional enhancers

As a concrete test of our ability to use SCRMshaw to predict functional enhancers, we assayed a subset of SCRMshaw predictions by reporter gene analysis in transgenic *D. melanogaster*. Our previous studies have shown a high success rate for such assays, ranging from 65% through >80%, depending on the species and training sets used (Kantorovitz et al., 2009; Kazemian et al., 2011; Kazemian et al., 2014; Schember and Halfon, 2021; Suryamohan et al., 2016).

**Figure 5.**
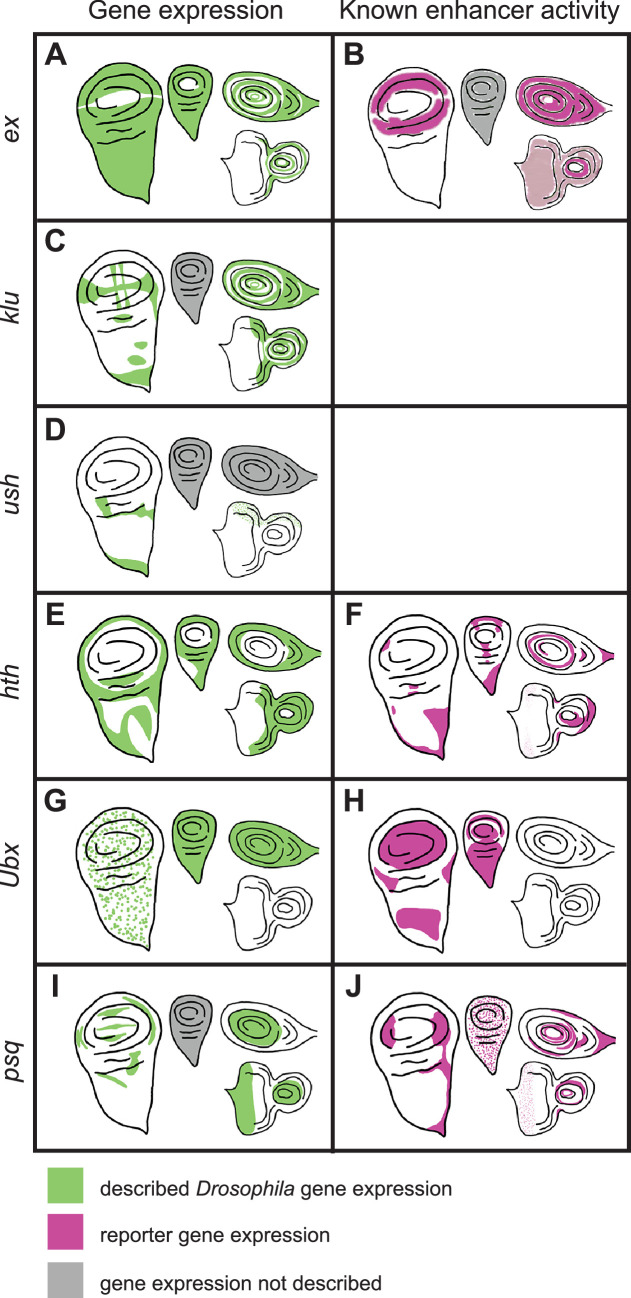
Previously described gene expression and enhancer activity for select *D. melanogaster* sequences predicted by SCRMshaw. The left-hand column shows native *D. melanogaster* gene expression in imaginal discs (green), while the right-hand column shows described enhancer activity (magenta). Gray shading indicates that expression has not been described. Moving clockwise from the left side of each panel are the wing, haltere, leg, and eye-antennal discs. The enhancers whose activities are described in the table are: (B) *ex_BCDE* (Wang and Baker, 2018), (F) *hth_GMR46D04* (Jory et al., 2012), (H) *Ubx_GMR39A02* (Jory et al., 2012), (J) *psq_GMR41E12* (Jory et al., 2012).

We focused our in vivo validation experiments on SCRMshaw predictions made using the *mapping2.wing, haltere_disc,* and *disc.mapping2* training sets and a set of four species we had previously shown to be amenable to SCRMshaw prediction: *D. melanogaster*, *A. aegypti*, *T. castaneum*, and *A. mellifera*. The imaginal discs are well-established tissues for investigating gene regulation in *D. melanogaster* (although see Discussion for a caveat on using imaginal discs for evaluating enhancer activities in a cross-species setting), and the chosen training sets all gave significant results in the open-chromatin comparisons discussed above. We selected six sets of putative enhancers for testing (Table 3, Table 4). For the first three sets, we selected sequences where we had predictions in each of the orthologous loci for *D. melanogaster, T. castaneum,* and *A. mellifera* (*A. aegypti* was not considered for these sets), and where the *D. melanogaster* prediction mapped near a gene expressed in the wing imaginal discs (Figure 5). These predictions were conducted using SCRMshaw’s IMM scoring method only, with post-processing performed using the original method described in Asma and Halfon, 2019, and the Amel_4.5 version of the *A. mellifera* genome. For the second three sets, we chose sequences where we had predictions in orthologous loci for at least three of the four species and where the *D. melanogaster* sequence had previously been tested in a reporter gene assay and was known to be active in the relevant imaginal discs (Figure 5). Imposing this latter criterion allowed us to leverage existing knowledge and reduce the necessary amount of in vivo testing for each set of predictions, enabling us to test a larger set of sequences overall. For this second set of three, we used all three SCRMshaw scoring methods with a revised post-processing algorithm (see Methods), and the Amel_Hav3.1 *A. mellifera* genome. For all six sets, predictions were chosen for testing based on high SCRMshaw scores and overlap with open-chromatin data (where available). We also considered the position of each prediction within the locus (i.e. first intron, downstream intergenic region, etc.), favoring sequences where position was maintained among the orthologs. Selected sequences were cloned into a cross-species compatible reporter vector (Lai et al., 2018; Deem et al., 2024), and reporter gene activity was visualized either directly or by using the lineage tracing system G-TRACE (Evans et al., 2009). In the latter, enhancer activity is visualized through two reporters; the first reporter visualizes the direct enhancer activity while expression of the second reporter is induced and maintained in all cells that descend from a cell in which the enhancer is initially active, even if activity subsequently shuts off.

**Table 3.** Gene loci chosen for in vivo validation.

*D. melanogaster*			*T. castaneum*		*A. mellifera*	*A. aegypti*
**Name**	**Symbol**	**FlyBaseID**	**RefSeq**	**iBeetleBase**	**RefSeq**	**VectorBase**
*expanded*	*ex*	FBgn0004583	*gene-LOC657053*	TC012545	*gene-LOC551519*	AAEL001437
*u-shaped*	*ush*	FBgn0003963	*gene-LOC659918*	TC013689	*gene-LOC100577801*	AAEL020615
*klumpfuss*	*klu*	FBgn0013469	*gene-LOC103312803*	TC002783	*gene-LOC100577692*	AAEL013544
*homothorax*	*hth*	FBgn0001235	*gene-Hth*	TC008629	*gene-LOC552079*	AAEL011643
*pipsqueak*	*psq*	FBgn0263102	*gene-LOC660343*	TC003349	*gene-psq*	AAEL021255
*Ultrabithorax*	*Ubx*	FBgn0003944	*gene-Ubx*	TC000903	*gene-ubx*	AAEL014032

**Table 4.** SCRMshaw predictions chosen for in vivo validation.

	Coordinates	Max. score	Training set(s)	Method
**Set 1**				
***T. castaneum***				
Tc_ex_9p0	NC_007424.3:11221530..11222170	9.04	mapping2.wing	imm
Tc_ush_6p8	NC_007420.3:5968840..5969370	6.84	haltere_disc	imm
Tc_klu_8p6	NC_007418.3:10416700..10417300	8.59	mapping2.wing	imm
***D. melanogaster***				
Dm_ex_20p0	2L:442110..442810	20.04	mapping2.wing	imm
Dm_klu_16p1	3L:10991040..10991700	16.06	mapping2.wing	imm
Dm_ush_16p4	2L:531250..532060	16.40	mapping2.wing	imm
***A. mellifera***				
Am_ex_20p3	NC_007075.3:7545750..7546440	20.36	mapping2.wing	imm
Am_klu_20p2	NC_007070.3:720220..720870	20.25	mapping2.wing	imm
Am_ush_20p8	NC_007080.3:10609550..10610200	20.80	haltere_disc	imm
**Set 2**				
***T. castaneum***				
Tc_hth_15p5	NC_007422.5:13408990..13409850	15.52	mapping2.wing	hexmcd,imm,pac
Tc_psq_19p7	NC_007418.3:1805000..1806250	19.71	haltere_disc, mapping2.wing	hexmcd,imm,pac
Tc_Ubx_19p9	NC_007417.3:8137250..8138000	19.32	haltere_disc	hexmcd
Tc_Ubx_17p4	NC_007417.3:8153250..8154030	19.95	mapping2.wing	hexmcd,imm
***A. aegypti***				
Aa_hth_35p9	1:149733960..149734710	35.96	mapping2.wing	imm
Aa_psq_21p5	2:228190750..228191640	21.50	disc.mapping2	hexmcd,imm
Aa_Ubx_26p0	1:309747490..309748390	26.08	mapping2.wing	imm
***A. mellifera***				
Am_psq_29p2	NC_007078.3:10712000..10712750	29.26	mapping2.wing	hexmcd
Am_Ubx_37p2	NC_007085.3:2921250..2922250	37.18	haltere_disc, mapping2.wing	hexmcd,imm
Am_Ubx_0p39	NC_007085.3:2967750..2968250	0.39	mapping2.wing	pac

#### ex

**Figure 6.**
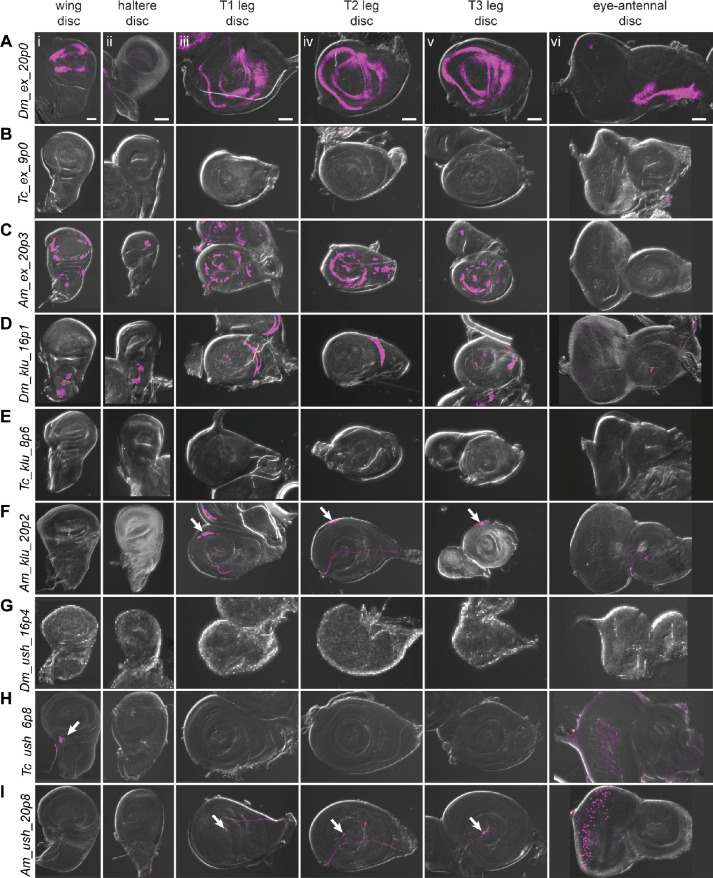
Reporter gene expression for tested *ex*, *klu*, and *ush* predicted enhancer sequences. Each row shows expression for the indicated construct in (i) wing discs, (ii) haltere discs, (iii) T1 (prothoracic) leg discs, (iv) T2 (mesothoracic) leg discs, (v) T3 (metathoracic) leg discs, and (vi) eye-antennal discs (with eye portion to the left). Positive results were obtained by the enhancers associated with the *ex* locus of *D. melanogaster* (wing, legs, antenna, A) and *A*. mellifera (wing, haltere, legs, C); the *klu* locus of *D. melanogaster* (wing, haltere, legs, D) and *A*. *mellifera* (legs, arrows in F); the *ush* locus of *T. castaneum* (wing, arrow, H (i); eye, H (vi)) and *A*. *mellifera* (legs, arrows I (iii, iv, v); eye, I (vi)). Enhancer activities were visualized by UAS-tdTomato that was included in the reporter construct. Scale bar is 50 µm for each column.

The *expanded (ex*) gene plays a crucial role in tissue growth control, including wings (Boedigheimer and Laughon, 1993; Wang and Baker, 2015; Wang and Baker, 2018). There was a single prediction in the *D. melanogaster ex* locus, *Dm_ex_20p0*, which falls within an open chromatin region of the third intron (Figure 6—figure supplement 1), and which comprises an untested subsequence of a longer sequence that acts as an imaginal disc enhancer (Figure 5B; Wang and Baker, 2018). This sequence drove reporter activity in the wing, leg, and antennal discs (Figure 6A). The *T. castaneum* genome also had only one prediction for the *ex* locus, *Tc_ex_9p0*, which overlaps well with a FAIRE-seq peak (Figure 6—figure supplement 1) within the second intron. Although no imaginal disc activity was observed (Figure 6B), *Tc_ex_9p0-*driven reporter gene expression was observed during late pupal stages in the legs (Figure 6—figure supplement 2G). The *A. mellifera* genome (v4.5) had one prediction, *Am_ex_20p3*, within the fourth intron of the *ex* locus (Figure 6—figure supplement 1; however, note that subsequent prediction using the updated Amel_Hav3.1 genome and revised SCRMshaw post-processing yielded additional predictions in this locus). *Am_ex_20p3* drove active but variable expression in both the pouch and notum regions of the wing imaginal disc (Figure 6C). Although often significantly limited to a small number of cells, the pouch expression of *Am_ex_20p3* was similar in pattern to that seen with *Dm_ex_20p0* (Figure 6A). Weak and inconsistent expression in the pouch region of the haltere discs was also observed. In addition, *Am_ex_20p3* drove expression in the leg discs (Figure 6C).

**Figure 6—figure supplement 1.**
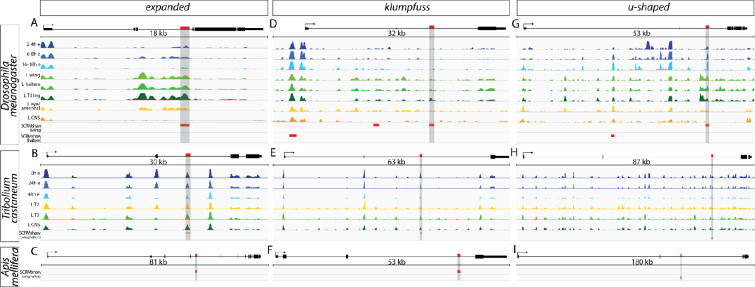
Open chromatin data for predictions in the *ex, klu,* and *ush* loci. SCRMshaw predictions (red bars) are shown for *D. melanogaster* (**A, D, G**), *T. castaneum* (**B, E, H**), and *A. mellifera* (**C, F, I**) at the *ex* (**A–C**), *klu* (**D–F**), and *ush* (**G–I**) loci. For *D. melanogaster* and *T. castaneum*, open chromatin profiles are also indicated, for a variety of tissues and timepoints (see text). Vertical gray bars highlight regions chosen for in vivo validation.

**Figure 6—figure supplement 2.**
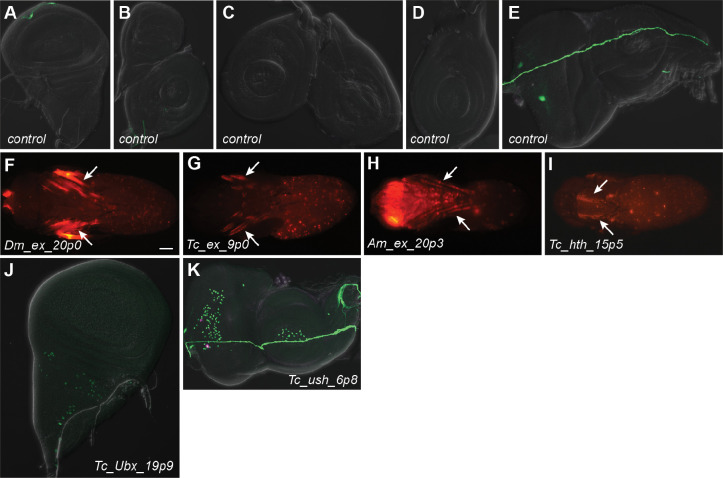
Additional expression observed in selected transgenic reporter lines. (**A–E**) Control lines using a regulatory-inactive mock enhancer sequence demonstrate that there is no default expression in the imaginal discs. (**A**) Wing disc, (**B**) haltere disc, (**C, D**) leg discs, (**E**) eye-antennal disc. (**F–I**) Pupal expression. Arrows indicate expression in the legs (**F–H**) or notum (**I**). For *T. castaneum enhancer Tc_ex_9p0*, expression was observed in pupal legs but not larval leg imaginal discs (G, compare with Figure 6B). (**J**) Expression can be observed in migrating adepithelial cells of the wing disc in *Tc_Ubx_19p9*. (**K**) Expression can be seen in the peripodial membrane surrounding the eye-antennal disc in several lines, including *Tc_ush_6p8* (see also Figures 6 and 7).

#### klu

*Klumpfuss* (*klu*) encodes a zinc finger protein important for proper tissue specification and differentiation (Klein and Campos-Ortega, 1997). The *D. melanogaster* genome had three predictions for the *klu* locus (Figure 6—figure supplement 1). We selected *Dm_klu_16p1*, which overlaps a region of open chromatin within the second intron present in most imaginal discs (most distinct in the T3 leg disc; Figure 6—figure supplement 1). *Dm_klu_16p1* drove active expression in the notum regions of the wing and haltere discs and in the leg discs (Figure 6D). *Tc_klu_8p6* was the only prediction for the *T. castaneum klu* locus. It falls within the second intron, similar to *D. melanogaster’s klu* prediction *Dm_klu_161p1* (Figure 6—figure supplement 1), but lacked enhancer activity (Figure 6E). *A. mellifera* had only one prediction for the *klu* locus (*Am_klu_20p2*) (Figure 6—figure supplement 1). We included this prediction for validation even though its location in the third intron does not match with that in the other two species. *Am_klu_20p2* drove weak expression in the most proximal part of the leg discs (Figure 6F, arrows), but no activity was observed in the other imaginal discs. (Note that additional predictions were subsequently produced with our revised post-processing algorithm and the updated Amel_Hav3.1 genome, for both the *T. castaneum* and *A. mellifera klu* loci.)

#### ush

Our final choice from our first set for in vivo validation was *u-shaped* (*ush*), which encodes a transcription factor with described imaginal disc expression (Buchberger et al., 2021; Cubadda et al., 1997; Tomoyasu et al., 2000). The *D. melanogaster* genome had two predictions for the *ush* locus (three when using the updated post-processing algorithm) (Figure 6—figure supplement 1). We tested *Dm_ush_16p4*, but did not observe any larval disc activity (Figure 6G). *Tc_ush_6p8* and *Am_ush_20p8* were the only predictions for *T. castaneum* and *A. mellifera*, respectively, both located in the second intron (again, additional predictions are found using updated methods and genome versions). *Tc_ush_6p8* had expression in the wing disc as well as weak activity in the peripodial membrane of the eye-antennal disc (Figure 6H, Figure 6—figure supplement 2K). *Am_ush_20p8* displayed active expression in the peripodial membrane surrounding the eye disc, as well as in a single cell, or small subset of cells, located at the center of each leg disc (Figure 6I, arrows).

#### hth

**Figure 7.**
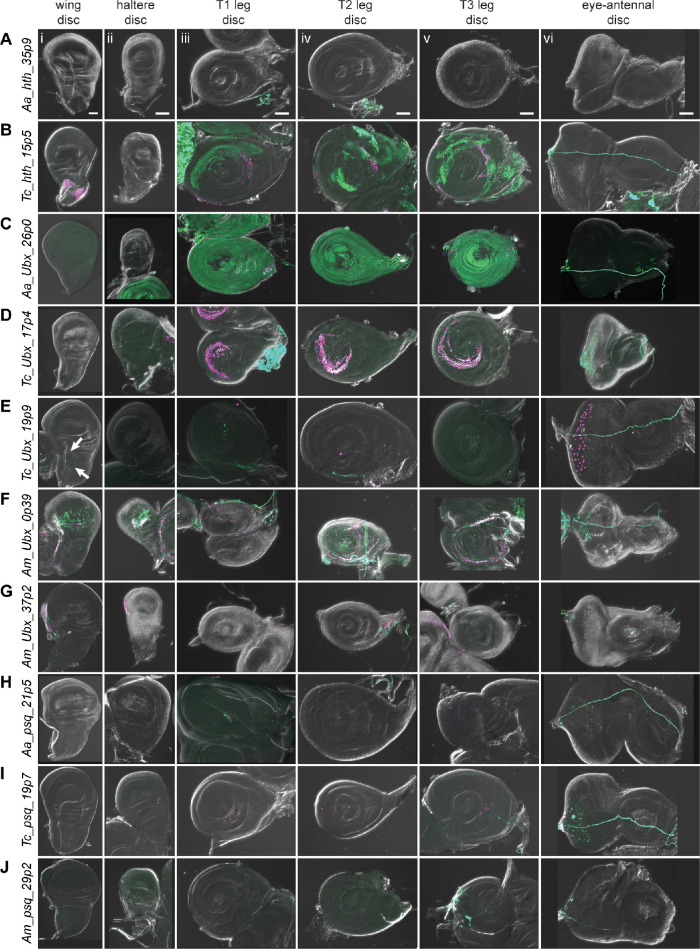
Reporter gene expression for tested *hth*, *Ubx*, and *psq* predicted enhancer sequences. Each row shows expression for the indicated construct in (i) wing discs, (ii) haltere discs, (iii) T1 (prothoracic) leg discs, (iv) T2 (mesothoracic) leg discs, (v) T3 (metathoracic) leg discs, and (vi) eye-antennal discs (with eye portion to the left). Positive results were obtained by the enhancers associated with the *hth* locus of *T. castaneum* (the notum portion of the wing disc, legs, B); the *Ubx* locus of *A. aegypti* (legs, C), *T. castaneum* (legs, D) (myoblast cells in the wing disc, arrows, E (i); legs, eye, E (iii–vi)), and *A. mellifera* (wing, haltere, legs, F) (wing, haltere, G); the *psq* locus of *T. castaneum* (legs, eye, I). Enhancer activities were detected by the G-TRACE system; magenta represents direct enhancer activity detected by dsRed expression, while green indicates lineage-based GFP expression. Scale bar is 50 µm for each column.

SCRMshaw predicted 10 enhancers in the locus of the *D. melanogaster* Hox cofactor *homothorax* (*hth*). One of the two top-scoring predictions, *Dm_hth_30p1*, located in the fifth intron, overlapped the known *hth_GMR46D04* enhancer, which has activity in all of the larval imaginal discs (Figure 5F, Figure 7—figure supplement 1; Jory et al., 2012). *A. aegypti* had a single prediction in the orthologous *hth* locus, *Aa_hth_35p9*, located in the first intron (Figure 7—figure supplement 1). This sequence failed to display activity in imaginal discs (Figure 7A).

**Figure 7—figure supplement 1.**
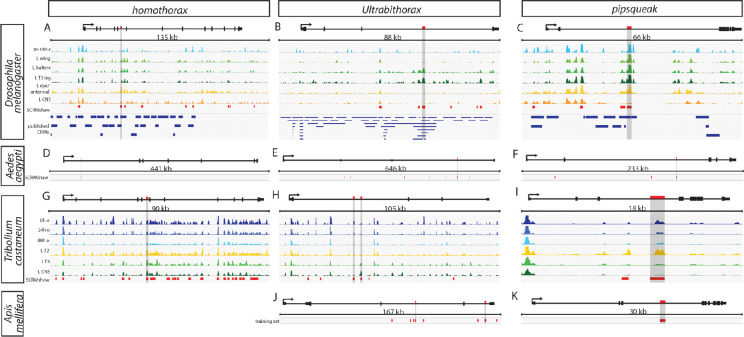
Open chromatin data for predictions in the *hth, Ubx,* and *psq* loci. SCRMshaw predictions (red bars) are shown for *D. melanogaster* (**A–C**), *A. aegypti* (**D–F**), *T. castaneum* (**G–I**), and *A. mellifera* (**J, K**) at the *hth* (**A, D, G**), *Ubx* (**B, E, H, J**), and *psq* (**C, F, I, K**) loci. For *D. melanogaster* and *T. castaneum*, open chromatin profiles are also indicated, for a variety of tissues and timepoints (see text). Blue bars indicate the positions of known enhancers. Vertical gray bars highlight regions chosen for in vivo validation.

**Figure 7—figure supplement 2.**
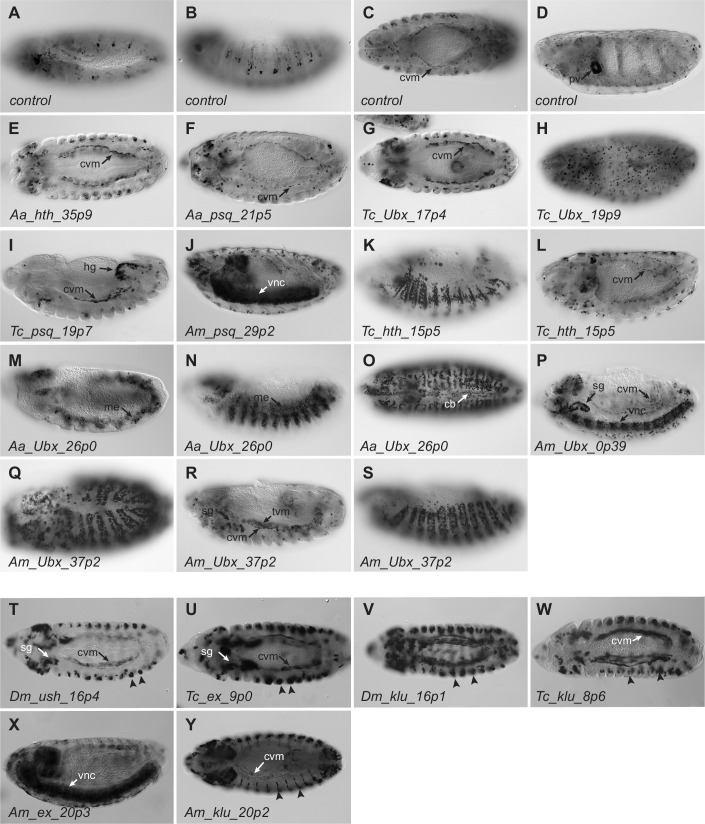
Reporter gene expression observed in embryos. Panels (A–S) are putative enhancers cloned into piggyPhiGUGd and crossed to G-TRACE. Panels (T–Y) have the putative enhancer inserted into piggyPhiGUGd-TomatoI. All embryos are stained using anti-dsRed antibodies and the ABC-HRP kit and shown with anterior to the left. (**A–D**) A non-enhancer control sequence was inserted into piggyPhiGUGd to serve as a control for vector-related expression. Limited segmentally repeated expression is observed starting around stage 11 (**A**) and can be observed in the epidermis and/or peripheral nervous system at stage 14 (**B**). (**C**) Expression is observed in the caudal (longitudinal) visceral mesoderm (arrow, ‘cvm’). (**D**) In older embryos, strong expression is observed in the proventriculus (‘pv’) as well as in migrating hemocytes (individual cells observed throughout the embryo). This ‘default’ expression pattern was also observed in enhancer lines *Aa_hth_35p9* (**E**), *Aa_psq_21p5* (**F**), *Tc_Ubx_17p4* (**G**), and *Tc_Ubx_19p9* (**H**). Note that not all stages are shown for each of these genotypes, as expression was highly similar for each line at all stages. While this expression was seen in the remaining reporter lines too, additional activity, which may therefore represent specific enhancer activity, was also observed. (**I**) *Tc_psq_19p7* had prominent hindgut (‘hg’) expression. (**J**) *Am_psq_29p2* had strong activity in the ventral nerve cord (‘vnc’). (**K**) *Tc_hth_15p5* displayed epidermal expression following germ band retraction; note that the caudal visceral mesoderm expression can also be observed (**L**). (**M–O**) *Aa_Ubx_26p0* had reporter gene expression throughout the mesoderm (‘me’) starting around stage 10 (**M**) and persisting throughout embryogenesis. ‘cb’, cardioblasts. (**P**) *Am_Ubx_0p39*, in addition to the default visceral muscle expression, was active in the ventral nerve cord (‘vnc’) and salivary glands (‘sg’). (**Q–S**) *Am_Ubx_37* p2 showed expression in the epidermis from stage 11 on, and also in the salivary glands. (**T–Y**) Although we do not have a no-enhancer control line for piggyPhiGUGd-dTomatoI, all lines generated using this vector had reporter gene activity in the caudal visceral mesoderm (‘cvm’), the salivary glands (‘sg’), and in segmentally repeated epidermal cells (arrowheads). We therefore view this expression as possible vector-specific expression (not all lines shown). However, two lines had additional, unique activity: *Am_ex_20p3* (**X**) was active in the ventral nerve cord, and *Am_klu_20p2* (**Y**) was active in narrow stripes in the epidermis (arrowheads). These activities may therefore be enhancer-specific.

*T. castaneum* had 23 *hth*-locus predictions. We selected a high-scoring (albeit not the highest-scoring) sequence, *Tc_hth_15p5*, due to the similarity of its position to that of the *D. melanogaster* enhancer—in the fifth intron—and overlapping FAIRE peak (Figure 7—figure supplement 1; Lai et al., 2018). The *Tc_hth_15p5* reporter showed activity in the presumptive notum region of the wing disc and in the proximal region of the leg discs, resembling both the endogenous *D. melanogaster hth* expression pattern and that of the *GMR46D04* enhancer (Figure 7B, compare with Figure 5F). No enhancer activity was observed in the haltere or eye-antennal discs (Figure 7B). In the pupal stage, *Tc_hth_15p5* exhibited active expression along the thorax, corresponding to adult *hth* expression in *D. melanogaster* (Figure 7—figure supplement 2, arrows) (Aldaz et al., 2005). No *hth* predictions were obtained for *A. mellifera*.

#### Ubx

The classic homeotic gene *Ultrabithorax* (*Ubx*) regulates tissue identity in the thoracic and abdominal segments (Lewis, 1978). Of six SCRMshaw predictions in the *D. melanogaster Ubx* locus, we noted that one, *Dm_Ubx_36p1*, overlaps a cluster of known enhancers in the third intron centered on *Ubx_GMR39A02* and *Ubx_abx6.8* (Figure 7—figure supplement 1). *Ubx_GMR39A02* mirrors the haltere activity of *Ubx*, but also displays ectopic activity in the pouch and notum regions of the wing disc (Figure 5H; Jory et al., 2012). Similarly, *Ubx_abx6.8* also drives both native and ectopic expression in the imaginal discs (Simon et al., 1990).

There were eight predictions in the *Ubx* locus of the *A. aegypti* genome (Figure 7—figure supplement 1). We selected sequence *Aa_Ubx_26p0*, as it was both the highest scoring prediction and was in the third intron, similar to its putative *D. melanogaster* counterparts. Although direct reporter expression from *Aa_Ubx_26p0* was too weak to observe directly, use of the lineage-tracing G-TRACE system confirmed widespread activity in all leg discs (Figure 7C).

We chose two *T. castaneum* sequences for testing, out of 10 predictions: *Tc_Ubx_17p4*, which overlapped well with an accessible chromatin region in the third thoracic epidermal tissue and the central nervous system (Lai et al., 2018), and *Tc_Ubx_19p9*, which had the highest local prediction score but only overlapped with a chromatin region accessible predominantly during early embryogenesis (Figure 7—figure supplement 1). *Tc_Ubx_17p4* displayed activity in all three leg discs (Figure 7D). The expression in T2 and T3 leg discs corresponds to endogenous *Ubx* expression, whereas T1 leg disc expression appears to be ectopic. *Tc_Ubx_19p9,* predicted using the ‘haltere’ training set, did not drive clear expression in the haltere disc but did drive G-TRACE expression in the wing disc in the adepithelial adult myoblast cells, as well as direct reporter expression in the peripodial membrane of the eye disc. Very limited expression was also observed in the leg discs when using G-TRACE (Figure 7E, Figure 6—figure supplement 2J).

The *A. mellifera* genome had 11 predictions at the *Ubx* locus (Figure 7—figure supplement 1). *Am_Ubx_37p2* was chosen based on a high SCRMshaw score, although it falls within the fourth, rather than the third, intron. *Am_Ubx_0p39*, on the other hand, was in the corresponding third intron location. *Am_Ubx_0p39* had activity in the pouch region of the wing and haltere discs, as well as in the proximal region of leg discs (Figure 7F). *Am_Ubx_37p2* drove expression in specific portions of the wing and haltere discs (Figure 7G). Although *Ubx* is not expressed in the *D. melanogaster* wing disc (i.e. the forewing of the fly), wing activity has been observed previously with tested *Ubx* enhancer fragments (e.g. Jory et al., 2012). Moreover, *Ubx* is expressed in both the forewing and hindwing discs in honeybees, making the reporter expression we observed driven by the two predicted *A. mellifera* enhancers consistent with their potential native activities (Prasad et al., 2016).

#### psq

We chose *pipsqueak* (*psq*), a transcription factor involved in Polycomb group gene silencing (Huang et al., 2002), and its orthologous loci as the final targets for enhancer validation. There were four predicted *psq* enhancers in *D. melanogaster*, one of which, *Dm_psq_25p6,* overlaps known enhancer *psq_GMR41E12* (Figure 7—figure supplement 1). This enhancer is located within the second *psq* intron and drives expression in all imaginal discs (Figure 5J; Jory et al., 2012). The *A. aegypti* genome had four predictions for the *psq* locus (Figure 7—figure supplement 1); we chose *Aa_psq_21p5*, located in the second intron and with the highest local SCRMshaw score, for validation (Figure 7—figure supplement 1). However, *Aa_psq_21p5* did not have observable imaginal disc expression (Figure 7H).

*T. castaneum* had only two predictions for the *psq* locus, both within the third intron (Figure 7—figure supplement 1). *Tc_psq_19p7*, which was chosen for validation due to its higher score, drove expression in the eye discs and in a very limited number of cells in the leg discs (Figure 7I).

From the *A. mellifera* genome, we selected the only prediction for the *psq* locus, *Am_psq_29p2* (Figure 7—figure supplement 1). *Am_psq_29p2* reporter activity was negative in all tissues assayed (Figure 7J).

#### Embryonic activity

Although our SCRMshaw predictions were targeted toward imaginal disc activity, activity was also observed in embryos for many of the reporter lines (Figure 7—figure supplement 2). Analysis of this activity was complicated by the fact that our reporter lines, while not having any basal activity in imaginal discs (Figure 6—figure supplement 2A–E), displayed reporter gene expression in several tissues including hemocytes, caudal visceral mesoderm, and the proventriculus, even in the absence of a putative enhancer sequence (Figure 7—figure supplement 2A–D). Similar expression is seen in control embryos of the G-TRACE line alone, i.e., even in the absence of a Gal4 driver (data not shown), suggesting that the observed basal activity might be coming from the UAS construct. In those lines that had reporter gene expression in other tissues (Figure 7—figure supplement 2I–S, X, Y), most of that expression was not clearly associated with the expected endogenous expression of the predicted target gene, although the complex embryonic expression patterns of these genes make a definitive assessment difficult. Moreover, for most of the species, we do not currently know the expression patterns of either the gene in its native species (as opposed to the expression of its *Drosophila* ortholog) or the expression patterns of other nearby potential target genes. Further analysis, including additional control experiments, use of different reporter vectors, and assessment of gene expression patterns in each of the relevant species will be necessary before drawing final conclusions as to embryonic enhancer activity.

### An insect regulatory annotation resource

Taken together, the results from our simulations, our comparisons to open chromatin regions, and our in vivo validation experiments demonstrate that SCRMshaw is remarkably effective at predicting regulatory sequences across a wide range of insect species. To facilitate access to our SCRMshaw-based regulatory annotations, we created a database with the results from our predictions using all training sets and all completed species. This database, which is freely accessible as part of the REDfly insect regulatory annotation site (Keränen et al., 2022, http://redfly.ccr.buffalo.edu), contains processed final prediction data and can be searched and filtered by gene, *D. melanogaster* ortholog, training set, enhancer location, and various other criteria. We will continue to add to this database as additional species and training sets are run through our annotation pipeline.

## Discussion

The resource we introduce here is part of an ongoing effort to provide an initial regulatory annotation for all sequenced insects. These annotations are not complete, as we currently lack well-curated training sets for many tissues, including key embryonic tissues such as the central nervous system and many non-embryonic tissues. We aim to generate the necessary additional training sets over time, and add these new annotations to those presented here, along with comprehensive annotation for additional species. As a predictive pipeline, SCRMshaw is subject to the usual tradeoffs of sensitivity versus specificity in generating results. Sensitivity is difficult to assess, as there is no ‘complete’ known set of enhancers for any organism, and it is currently impossible to make an accurate estimate of what the yield should be for any of our training models. Similarly, without comprehensive in vivo testing using multiple conditions and methods, an accurate false-positive rate cannot be computed. However, we presented here several lines of evidence demonstrating that true positives significantly outweigh false positives: we non-randomly predict multiple enhancers for specific subsets of loci; we predict enhancers in orthologous loci at a rate significantly higher than random expectation; our predictions are highly enriched for regions of accessible chromatin; and we achieve a high rate of validation using in vivo reporter gene assays.

### Validation success rates

**Figure 8.**
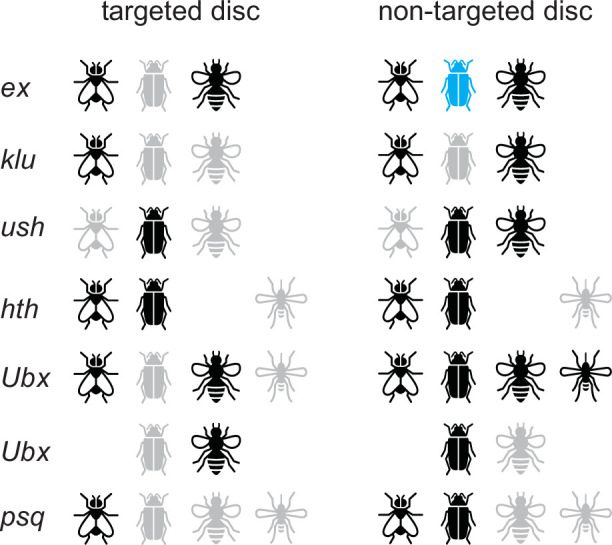
Summary of in vivo validation results. Results are shown for *D. melanogaster, T. castaneum, A. mellifera,* and *A. aegypti.* Black, positive for expression; gray, negative for expression; blue, expression observed in pupae but not in larvae.

The results from the in vivo validation experiments are summarized in Figure 8. Overall, 17/22 (77%) of tested sequences revealed imaginal disc activity, consistent with our previous SCRMshaw success rates. 59% of these (10/17), or 45% of the total (10/22), had the correct target specificity of wing and/or haltere discs. This is again consistent with previous SCRMshaw experience, which shows that functional enhancers are predicted at a higher success rate than enhancers with specific targeted activity (Kantorovitz et al., 2009; Kazemian et al., 2011).

Image credits: The insect silhouettes were obtained from The Noun Project (https://thenounproject.com/), artist Georgiana Ionescu, under a Creative Commons CC-BY 3.0 license.

These numbers may in fact underestimate the success rate of our predictions, for several reasons. One, all of the testing was performed in transgenic *D. melanogaster*, despite the putative enhancers being from three additional species. Reduced efficiency of certain transcription factor or cofactor binding, reduced enhancer-promoter compatibility, or other species-specific differences may lead to elevated false-negative results. Furthermore, the unique imaginal disc mode of adult epithelial development in *D. melanogaster* (Cohen, 1993; Svácha, 1992) might have prevented some enhancers of other species from working properly in *D. melanogaster* imaginal discs, likely producing additional false-negative results. Evaluating enhancer activities in the native species will allow us to address the degree of false negatives produced by the cross-species setting. Two, predictions for *ex, klu*, and *ush* were made using a less-effective SCRMshaw post-processing algorithm and, for *A. mellifera*, a less-complete genome build. This may have led to suboptimal candidate enhancer selection. Three, it is possible that some of our predicted enhancers act as silencers rather than enhancers. Silencers, which attenuate rather than promote gene expression, are less well understood than enhancers, but in at least some instances have identical sequence characteristics (and in fact can act simultaneously as enhancers in some tissues and silencers in others) (Halfon, 2020; Segert et al., 2021). Our reporter gene assay was not designed to detect silencers, which would therefore appear as false-positive predictions. Indeed, an intriguing possibility is that some of our sequences that had reporter gene activity in non-targeted discs (e.g. leg discs) act as enhancers in those tissues but as silencers in the targeted wing discs. Alternatively, identified enhancers that drove expression in multiple imaginal discs may simply represent pleiotropic enhancers. Enhancer pleiotropy is not uncommon (Laiker and Frankel, 2022; Sabarís et al., 2019), and moreover, there is overlap in the enhancer content of our training sets for the various discs. There are many gene expression and developmental similarities among the discs, and likely significant regulatory overlap; chromatin profiling experiments have found that wing, leg, and eye discs share the majority of their regions of accessible chromatin (Lai et al., 2018; McKay and Lieb, 2013). Additional experiments will be necessary to distinguish between pleiotropic enhancers, dual-function silencers/enhancers, and other possibilities.

### Redundant enhancers

Almost all of our training sets predicted multiple enhancers in certain loci at rates exceeding random expectation, consistent with the noted prevalence of redundant or ‘shadow’ enhancers in numerous plant and animal species (reviewed by Kvon et al., 2021). The few training sets that did not may reflect types of tissues or regulatory processes for which having shadow enhancers is unusual, or may be indicative of poorly performing training sets. Indeed, the sole training set that did not suggest the presence of shadow enhancers in either mosquito species, *adult_PNS*, also performed poorly by other metrics, such as failing to produce predictions in common over multiple species and scoring ‘poor’ using our *pCRM_eval* training set evaluation tool (Asma and Halfon, 2019).

Although the evolutionary origins of shadow enhancers are not well understood, several studies suggest that at least one important contribution of shadow enhancers is to provide phenotypic robustness during development, particularly during stress conditions (Frankel et al., 2010; Perry et al., 2010; Perry et al., 2011; Osterwalder et al., 2018; Antosova et al., 2016; Sagai et al., 2017). In some cases, a mechanistic basis for this robustness has been suggested by the finding that different groups of transcription factors act on individual members of a shadow enhancer set (Waymack et al., 2020; Cannavò et al., 2016). Further support for this idea can be found in the fact that shadow enhancers appear to have limited sequence similarity (Waymack et al., 2020; Barth et al., 2020). However, our successes with SCRMshaw have demonstrated that enhancers can have little overt sequence similarity but still rely on the same underlying subsequence (and presumably, transcription factor binding) model. As the potential shadow enhancers we identify are predicted using the same training set, they are likely to be functioning using similar, rather than independent, regulatory mechanisms. Thus, shadow enhancers appear to come in at least two flavors: those that use similar mechanisms, such as those identified here, and those that use different mechanisms, such as the *Krüppel* enhancers studied by Waymack et al., 2020.

### Enhancer evolution at large divergence distances

SCRMshaw’s ability to find putatively ‘orthologous’ enhancers—i.e., enhancers in orthologous loci predicted using the same regulatory model—not only substantiates the non-random nature of our prediction approach, but also opens the door to exciting studies of enhancer evolution over large divergence ranges. We previously illustrated the power of such an approach with an analysis of a small number of enhancers in just two to three highly diverged species (Kazemian et al., 2014). However, the greatly increased number of species for which we now have regulatory predictions should allow us to follow the evolution of specific enhancers over their entire divergence range. Although the number of common loci tails off rapidly as the number of species considered increases, we suspect that this small (albeit statistically significant) number understates the true results. For one, only a limited number of species have been evaluated for common predictions so far, and these are not evenly distributed along the phylogenetic spectrum of sequenced species. Also, our analysis depends wholly on the presence of recognized *D. melanogaster* orthologous genes to define the common loci. This in turn is dependent on several factors, including the sensitivity and accuracy of our ortholog-calling pipeline, the reliability of the protein annotations we use in that pipeline, and on accurate target gene assignments. Each of these has known sources of error. For example, our ortholog-calling pipeline currently does not disambiguate multiple paralogs (see Methods), and ortholog detection has been shown to be sensitive to the method used for generating protein annotation, in particular when different annotation methods have been applied to different species in the comparison (Weisman et al., 2022). Moreover, our target gene assignments are presently based solely on closest-gene relationships, a method which is known to mis-assign a fair number of enhancer-gene target pairings (e.g. Sanyal et al., 2012; Hafez et al., 2017; Chua et al., 2022; Qin et al., 2022) and which does not take into account complexities in genome architecture such as nested genes, long non-coding RNAs, promoter competition, and the like (which are common in *D. melanogaster*, e.g. Crosby et al., 2015; Matthews et al., 2015). Developing effective and scalable solutions to these issues will be an important goal for future work.

As the number of species in our prediction database with fully mapped orthologs grows, it will become possible to ask increasingly sophisticated questions about the nature of enhancer evolution. For instance, we will be able to determine whether the numbers of putatively orthologous enhancer predictions follow phylogenetic relationships and degree of sequence divergence, and whether certain loci are only found in common for specific groups of species. Also in need of further investigation will be to determine whether sets of common enhancers are restricted to certain functional Gene Ontology categories, and how this might vary with phylogenetic grouping.

### Ongoing regulatory annotation

While effective, SCRMshaw still has various limitations and aspects in need of improvement. Errors in genome assembly and insufficient repeat masking both appear to contribute to overly long predictions (multiple kilobases) that are unlikely to represent individual single enhancers (HA and MSH, unpublished observations). Poor assembly, while not having major effects on SCRMshaw overall, can significantly affect results at specific loci, as can inaccurate gene annotation (Asma and Halfon, 2019). Also requiring further investigation is how to best combine and weight the scores from the three individual SCRMshaw scoring methods of IMM, hexMCD, and PAC-rc. Individual SCRMshaw predictions should therefore be treated as just that—predictions—and appropriate validation experiments are recommended for any sequences of interest. The regulatory annotations presented here represent initial ‘1.0’ versions of the regulatory genome. Like all genome annotations, these will continue to be revised as updated models become available, including addition of new training sets, confidence scores based on validation experiments, and improved genome builds. Nevertheless, the enrichment scores from our in silico experiments and the true-positive rates from our in vivo reporter gene experiments suggest that overall, true-positive rates for most training sets are between 50% and 85%, with most likely having success rates exceeding 70%. Note that even this lower bound of a 50% true-positive rate means that one out of every two predictions is correct—a more than acceptable rate to encourage follow-up experiments for predicted enhancers in organisms of interest. The catalog of predicted enhancers we introduce here, spanning 33 insect species and growing, should thus be a useful resource for both large-scale and small-scale studies of the insect regulatory genome.

## Methods

### Datasets

Sequence and annotation for *D. melanogaster* were obtained from FlyBase (Gramates et al., 2022). For other species, wherever possible, genome sequence (FASTA) and annotation (GFF) files were downloaded from NCBI at https://www.ncbi.nlm.nih.gov/datasets. Otherwise, the genome sequences and annotations were obtained from the primary literature or directly from the data generators (see Table 1 for references). Version numbers for genomes and annotations are provided in Table 1.

### Scripts

A permanent archive of this code can be found at Zenodo: https://doi.org/10.5281/zenodo.13821366. All scripts used for the analyses described in this paper can be found in the GitHub repository Asma_etal_2024_eLife at https://github.com/HalfonLab/Asma_etal_2024_eLife, (copy archived at Asma and Halfon, 2024).

### SCRMshaw

A detailed SCRMshaw protocol can be found at Asma et al., 2024.

Genome files were masked using Tandem Repeat Finder (Benson, 1999) using parameters 2 7 7 80 10 50 500 -m -h. Genome and annotation files were then assessed using our *preflight* script. Any sequence scaffolds not containing annotated genes were removed before passing the genome sequence to the main SCRMshaw program. SCRMshaw was run using the ‘HD’ version, which scans the genome with a 500 bp window sliding in 10 bp increments, as described in Asma and Halfon, 2019. The *--thiwt* parameter was set to 5000, and *--lb* to [0,10,20,30,…,240] for each of 25 instances, respectively. All other parameters were kept at default values.

SCRMshaw makes use of three underlying statistical models, described briefly in Kazemian et al., 2014, and in detail in Kantorovitz et al., 2009; Kazemian et al., 2011. *HexMCD* trains a fifth-order Markov chain on all 6-mers and their one-away mismatches in the training and background sequence sets, respectively, while *IMM* trains an interpolated Markov model that combines Markov chains of all orders from 0 to 5. For both of these methods, the score for a sequence is the log-likelihood ratio of the model trained on the training sequences divided by the model trained on the background sequences. *PAC-rc* quantifies the overrepresentation of 6-mers in the training versus background sets, assuming a Poisson distribution of word counts. Our previous work has shown that each method is effective, and each tends to have sensitivity for a different group of enhancers. Roughly three-quarters of all predicted enhancers were identified by just a single method (see Figure 2—source data 2), although there is a strong correlation between high-ranking predictions and prediction by more than one method. The best results are obtained from combining the output of all three methods.

SCRMshaw can be downloaded at https://github.com/HalfonLab/SCRMshaw_HD, (copy archived at Asma and Halfon, 2023).

### Post-processing

The top 5000 hits were extracted using scripts *Generate_top_N_SCRM-hits.pl* and *concatenatenatingOffsetResults.sh* and passed to *postProcessingScrmshawPipeline.py* with *-num* = ‘5000’ and -*topN* = ‘Median’. Post-processing was performed essentially as previously described (Kazemian and Halfon, 2019; Asma and Halfon, 2019), with the following modifications (Figure 1—figure supplement 1): before evaluating each 10 bp region, scores from each of the 25 individual SCRMshaw instances were assessed and any 500 bp window whose score was below the value of the 5000th ranked score was eliminated by having its score reset to zero (Figure 1—figure supplement 1B). The ‘elbow’ point of the SCRMshaw score curve of the 5000 top scores from each instance was then determined (Figure 1—figure supplement 1C), and any scores below the elbow point were reset to zero (Figure 1—figure supplement 1D; gray boxes). This is a key modification to our previous SCRMshawHD protocol (Asma and Halfon, 2019) and reduces the median prediction size by preventing concatenation of multiple adjacent low-scoring windows. Only after these two rounds of score evaluation were all windows grouped together (Figure 1—figure supplement 1E, F) and subjected to peak calling on 10 bp intervals (Figure 1—figure supplement 1G). Final ‘top predictions’ were then any peaks with an amplitude above the selected amplitude threshold (elbow point of amplitude curve, represented by a red dot in Figure 1—figure supplement 1H), following the peak-calling step.

All scripts are available at https://github.com/HalfonLab/.

### Orthology mapping

The final SCRMshaw output from the above steps was used as input to our orthology mapping pipeline. For each species, a FASTA-formatted file of all annotated proteins was downloaded from NCBI (https://www.ncbi.nlm.nih.gov/datasets). The annotated proteins from *D. melanogaster* plus each individual other species were used as input to *Orthologer* (Kuznetsov et al., 2023) to obtain the *Drosophila* ortholog for each protein (when existing). Our approach was designed to be minimally restrictive in that we did not enforce a one-to-one ortholog mapping; in cases of likely paralogs, we considered all of the paralogs as a potential result. Details on the orthology mapping protocol can be found in Asma et al., 2024.

### Evaluating the number of predictions per locus

For each training set, BEDTools ‘merge’ was used to remove any overlapping predictions (Quinlan and Hall, 2010). The results were then permuted 1000 times using BEDTools ‘shuffle’, with coding regions excluded. BEDTools ‘closest’ was used to assign upstream and downstream flanking genes to each of the permuted predictions, with the following parameters: the ‘-io’ flag was enabled to ignore any overlaps between predictions and genes, and ‘-D ref -id’ and ‘-D ref -iu’ were used to obtain the closest 5’ and 3’ genes (with respect to chromosome coordinates), respectively. A custom Python script, *checkSameLocus_ForSimulations.py* (see https://github.com/HalfonLab/Asma_etal_2024_eLife), was used to calculate the number of predictions per locus for both the real and permuted results. For this purpose, ‘locus’ was defined as the entire region between the left and right flanking genes for each SCRMshaw prediction; e.g., for a prediction between two genes in a head-to-tail orientation we take the region between the 3’ end of the upstream gene and the transcription start site of the downstream gene. If a prediction is within an intron, we define the locus as the entire span of the enclosing gene. If a prediction overlaps two genes, the locus is considered to be the entire span of the two genes. Average intergenic region sizes for the genomes were estimated using the distances between neighboring genes, discarding any nested or overlapping gene pairs, as provided by the SCRMshaw *preflight* script.

Significance was assessed by calculating the empirical p-value, defined as the number of real loci with a number of predictions greater than the maximum number obtained from the 1000 simulations, for each locus containing at least one SCRMshaw prediction.

### Evaluating the number of predictions in common across species

To determine the expected number of common predictions—i.e., predictions in orthologous loci—across species, we utilized SCRMshaw predictions for 16 species. Each set of predictions was sorted, merged, permuted, and mapped to new loci as described above for ‘Evaluating the number of predictions per locus’. The permuted predictions were used as input for the script ‘*checkSameLocus_ForSimulations_crossSpecies.py*’ (see https://github.com/HalfonLab/Asma_etal_2024_eLife). This script identifies the *Drosophila* orthologs of the nearest flanking genes for each species and calculates the number of common loci flanking the simulated SCRMshaw predictions for 5, 10, 11, 12, 13, 14, 15, and 16 species. For each species, the process was repeated for a total of 360 permutations. The mean and standard deviation of the permuted results were then used to calculate a z-score for each training set. We considered training sets with z-score ≥1.645 to be significant (p<0.05, not corrected for multiple testing).

### Overlap between SCRMshaw predictions and open chromatin regions

Open chromatin data were obtained from the following sources:

*D. melanogaster*: Data for *D. melanogaster* were downloaded from GEO and consisted of ATAC-seq and FAIRE-seq data from accessions GSE101827, GSE38727, and GSE118240. These assays were performed using blastoderm embryos, eye-antennal discs, wing discs, haltere discs, leg discs, third instar central nervous system, and wing, leg, and haltere pharate appendages (Bozek et al., 2019; McKay and Lieb, 2013; Jacobs et al., 2018).

*T. castaneum*: FAIRE-seq data for three stages of embryogenesis, larval central nervous system, and larval second and third thoracic epidermal tissues were downloaded from GEO (GSE104495) (Lai et al., 2018). The FAIRE profiles were remapped to the version 5.2 of the *T. castaneum* genome (Tcas5.2) for this study. The remapped FAIRE profiles are available on iBeetle-Base (https://ibeetle-base.uni-goettingen.de/, Dönitz et al., 2018).

*A. gambiae*: ATAC-seq data for adult midgut and salivary gland were downloaded from GEO (GSE152924) (Ruiz et al., 2021).

*D. plexippus, J. coenia, H. himera,* and *V. cardui*: ATAC-seq data for larval forewing and hindwing tissues at stage M5 were provided by Anyi Mazo-Vargas and Robert Reed (Mazo-Vargas et al., 2022).

The overlap between SCRMshaw predictions and open chromatin regions was determined using BEDTools ‘intersect’ with parameters -wa -u -f 0.1 such that sequences needed to overlap at least 10% of their length and were considered a single overlap in the event that more than one open chromatin peak overlapped a prediction.

To assess significance, the SCRMshaw predictions were permuted 500 times using BEDTools ‘shuffle’ and the overlaps with open chromatin regions assessed as above. The mean and standard deviation of the permuted results were then used to calculate a z-score for each training set. We also calculated a ‘fold enrichment’ score by dividing the observed number of overlapping regions by the expected number, to provide a sense of effect size in addition to statistical significance.

### Reporter constructs and transgenic *Drosophila*

Sequences for reporter gene analysis, including attL1 and attL2 sites, were synthesized de novo and cloned into pUC57 Kan-r (GenScript, Piscataway, NJ, USA) as entry vectors suitable for Gateway cloning (Katzen, 2007). Gateway LR recombination was then used to move the sequences into piggyPhiGUGd (*hth, Ubx,* and *psq* lines) and piggyPhiGUGd-TomatoI (*ex, klu,* and *ush* lines) (Deem et al., 2024) (these reporter vectors are available from the Drosophila Genomics Resource Center, https://dgrc.bio.indiana.edu). Transgenic flies were generated by BestGene (Chino Hills, CA, USA) using PhiC31 recombination and the attP2 third chromosome insertion site, and are available on request. piggyPhiGUGd lines were subsequently crossed to G-TRACE for visualizing enhancer activities (Evans et al., 2009).

For each construct, imaginal discs from at least six larvae were dissected and mounted for direct fluorescence visualization using a Zeiss Axio Imager M2 microscope with ApoTome 2. Embryos were fixed and stained using standard *Drosophila* methods using anti-dsRed (Clontech) and visualized using the ABC-HRP kit (VectorLabs, Newark, CA, USA).

### Database implementation

The SCRMshaw results database is implemented as part of REDfly (RRID:SCR_006790; Keränen et al., 2022), a MariaDB-based database hosted on a private OpenStack cloud infrastructure maintained by the University at Buffalo Center for Computational Research. As part of an ongoing transition of REDfly to a more modern software architecture, backend functions are implemented in Node.JS and Python, while frontend components utilize React.JS and Next.js. GraphQL is used as the query language. REDfly is licensed under a Creative Commons Attribution-NonCommercial-NoDerivatives License v4 International (CC BY-NC-ND 4.0) and its underlying source code under a GNU General Public License v3 (GNU GPL 3.0).

## Data Availability

Data generated in this study has been deposited at Dryad at https://doi.org/10.5061/dryad.3j9kd51t0 in both the standard SCRMshaw output format (modified BED) and in GFFv3 format. Data can also be obtained via the REDfly database at http://redfly.ccr.buffalo.edu. Software is available at https://github.com/HalfonLab/Asma_etal_2024_eLife, copy archived at Asma and Halfon, 2024. The following dataset was generated: AsmaH TiekeE DeemKD RahmatJ DongT HuangX TomoyasuY HalfonMS 2024Data from: Regulatory genome annotation for 33 insect speciesDryad Digital Repository10.5061/dryad.3j9kd51t0PMC1146967039392676 The following previously published datasets were used: JacobsJ AertsS 2018The transcription factor Grainyhead primes epithelial enhancers for spatiotemporal activation by displacing nucleosomes [ATAC-seq]NCBI Gene Expression OmnibusGSE10182710.1038/s41588-018-0140-xPMC603130729867222 McKayD LiebJD 2013A common set of DNA regulatory elements shapes *Drosophila* appendagesNCBI Gene Expression OmnibusGSE3872710.1016/j.devcel.2013.10.009PMC386652724229644 BozekM CortiniR StortiAE UnnerstallU GaulU GompelN 2019Genome-wide profiles of chromatin accessibility in spatially-restricted domains along the antero-posterior axis of *Drosophila* blastodermNCBI Gene Expression OmnibusGSE118240 LaiY DeemKD Borras-CastellsF SambraniN RudolfH SuryamohanK El-SherifE HalfonMS McKayDJ TomoyasuY 2017Enhancer identification and activity evaluation in the red flour beetle, Tribolium castaneumNCBI Gene Expression OmnibusGSE10449510.1242/dev.160663PMC1173665829540499 RuizJL Ranford-CartwrightLC Gómez-DíazE 2020The regulatory genome of the malaria vector Anopheles gambiae: integrating chromatin accessibility and gene expressionNCBI Gene Expression OmnibusGSE15292410.1093/nargab/lqaa113PMC809244733987532

## Additional files

MDAR checklist

## Appendix 1

**Appendix 1—key resources table:**

Reagent type (species) or resource	Designation	Source or reference	Identifiers	Additional information
Gene (*Drosophila melanogaster*)	expanded (ex)	FlyBase	FBgn0004583	
Gene (*D. melanogaster*)	u-shaped (ush)	FlyBase	FBgn0003963	
Gene (*D. melanogaster*)	klumpfuss (klu)	FlyBase	FBgn0013469	
Gene (*D. melanogaster*)	homothorax (hth)	FlyBase	FBgn0001235	
Gene (*D. melanogaster*)	pipsqueak (psq)	FlyBase	FBgn0263102	
Gene (*D. melanogaster*)	Ultrabithorax (Ubx)	FlyBase	FBgn0003944	
Gene (*Tribolium castaneum*)	gene-LOC657053	iBeetleBase	TC012545	
Gene (*T. castaneum*)	gene-LOC659918	iBeetleBase	TC013689	
Gene (*T. castaneum*)	gene-LOC103312803	iBeetleBase	TC002783	
Gene (*T. castaneum*)	gene-Hth	iBeetleBase	TC008629	
Gene (*T. castaneum*)	gene-LOC660343	iBeetleBase	TC003349	
Gene (*T. castaneum*)	gene-Ubx	iBeetleBase	TC000903	
Gene (*Apis mellifera*)	gene-LOC551519	RefSeq		
Gene (*A. mellifera*)	gene-LOC100577801	RefSeq		
Gene (*A. mellifera*)	gene-LOC100577692	RefSeq		
Gene (*A. mellifera*)	gene-LOC552079	RefSeq		
Gene (*A. mellifera*)	gene-psq	RefSeq		
Gene (*A. mellifera*)	gene-ubx	RefSeq		
Gene (*Aedes aegypti*)	AAEL001437	VectorBase		
Gene (*A. aegypti*)	AAEL020615	VectorBase		
Gene (*A. aegypti*)	AAEL013544	VectorBase		
Gene (*A. aegypti*)	AAEL011643	VectorBase		
Gene (*A. aegypti*)	AAEL021255	VectorBase		
Gene (*A. aegypti*)	AAEL014032	VectorBase		
Antibody	anti-dsRed (Rabbit polyclonal)	Clontech	Cat#632496	(1:500)
Recombinant DNA reagent	piggyPhiGUGd (plasmid)	Deem et al., 2024, PMID:38698030		
Recombinant DNA reagent	piggyPhiGUGd-TomatoI (plasmid)	Deem et al., 2024, PMID:38698030		
Sequence-based reagent	Dm_ex_20p0	This paper		TTCCCAGAACAAACTTGTGGGGGGTGATTAGGTTTGGCAACAAAATATTTTGCTAGTATTCCCTAATCATTTTTTTGAGTGAACCAAACTCGAAGAGCTCTACTCCCCTGGCCATCCACTTGTTGCCACTTCCATTCCAGCTTTGCGTCGACGACGTCGTCATTGATAGGCACTTATTCGGCCGCTGATGATTATTATGATATTGTAGCTGCTGCTGCTGTGTTGTGGATTCGATGCTGAGGTGCCTCTATTCCATGGCCTCCTTCAACCTGCCTGCCTGCTTTTTTCATAATTATTATTTTTCATCTTGCTGCTCTTCATTTTGTATGCAGGAATTCCAATTTTTCGTTCGATGAAGTGTGTGTTGATTTCGCTGTTGTTTTTTCTCTGCCTTCTCGAGCACCGCCGACATGCCCTTGGGCCCTTCTGCTTGGCTCGGGTCGGAGCTATGTAGCGCGGTCCGGTACCGGTCTCGTCTTCGAGCATCAGGCAATGGGCCTCTGACAACCTGACGTGTCGTCATCATCATCGTCTTCATCTGCTGGAGTCTCTGACTCTTGTTGATGTCAATGGGTTGCTTGTTTATTGCCTGACAAACTGACAGAAGTCTGGTCGGGGTCTCGATCCGATTTGAGCCCGATTTGGGACGCAAGAGGAGCGCTCCCTCTTGCATAGCCGAAAGTTCATTTAAAATTTTGAT
Sequence-based reagent	Dm_klu_16p1	This paper		GACCAGGCTGTTGCAGTTTCGTGTTGAAACCAGTTTGAATATATTTATTTTTATTTCCTGCGTCCCCTTCCCAATTTCTGTGGCCCTTTTAGGCGCCTCAGTTAGTCGGCAACGATAAGGCGGCAATGGTTTAATTTAGCTGCACCAGCGGCAGCAGCAGATGACGACAGGATCGTTGGGCCGGTCTACGTGCAACAGAAGTTGCTGCGCCGGCAGAAGCAACGGCAGCGGCAGCAACAGCAACAGCAAAACAAATGTGTCTGTATATCGCAGCTAAATTGACTTTGATCACGCGATCCCGAATCCCCCCCCCCATTTGGTCCGAGTTATATGGCCGATTCCAGGTTGCAGGCTCCAGGTTCTCGGGCGCGGCCTTTTGTGGCACAAACGGAAGTATGCTAAGCAACTTGTTGCTGCCGCAAAGGCAAAGCAGCAAAAGCAGCTGAAGGTGTATATTGCAAAAATAATTACATTTGATTGTAAAAGGCCAGCGTCTCTAGGCTGGGGACTCGAGATCGGACCTCGGCCTGCCAGAGAAAAATGTGCAACATGATTGCAGTTTACAGCCCCAGCAGCAGCAGCAGCAGCTAAAGCAGCAACAACAACAGCAGCAGCAGCAGCAAAACCAACAGATAAAATGCATTTACAATTGAATTTGAA
Sequence-based reagent	Dm_ush_16p4	This paper		GAATGTTGCTGCGGTGGCATGTTGTTGCTCGTCGAAGTTCAGCCGATGTTGCTGCTGCTGCTGCAGCTGCTGTTGCTGCCATTCCCACTATCAACCGATGGTAATCGAAGGAAGCGACATTTATGCAAATGCCAGGTTGTTTAATAAACGCAAATTATGAGCCCGGCAGCAACATGTTGCAGCAACAGTCGATGGCAGATTAGCGACATTCATACTTGCACTTGGGTCAATTTAAATTTGTGCAACAGTGGCAGCACGGCACGGCAGCAACTCCTTGCCGCAGCAGCAGCCGCTCCAGCAGCACATGAGATTGTGGGAGCAACAGGCAGCATTATTGTGTTCGGCCAAGATCGCAATTGATCAGTGTGTTGGTGCTGGTGTTGGTGTTGCAGTTGCAGTTGCAGTTGCAGTTGCAGTCGCTGTTGCTGTTGCTGTTGCTACCCGACGACAACAGTTGCTGCTGTGCTGGTGCTAGTGCTTGTGCTTGTGCTTGTGCTTGTGTTGCTGCTGATCAAGCGATCAAGCACCGCAGCCAAAAACAATCGGCGCTGAGCGTGCTCACACGAAATTTTCAAGTACTGCGACAATTTCCATGCCCCCAGCCGCTGCCTTGTTATCAGCGCGCCATGCAACAGCAACAGCGACTGCAGCACAGGCAACAGCAGCAACACATCTCAACAGTTGCATCAATTGCTCAACATTGAACTCTGGGCATGGGCCCAGACGATCACCCCTCCTGGGGACCCCCTTCGGTCGCCCCTGCCCCAGTCCCTTGTATCATTTGCACGTTTTTTAATTAAGACATCAAAA
Sequence-based reagent	Tc_ex_9p0	This paper		ATAGTTCTAAAGTTCTAAACTATTTGCAAATGTAAACAACGACCGACATTTTCAACATGTTCGGGTGTACGTCGCTTTGAATATGGAAAATGTGATTTTGTAGAGAAATTTGGTGGCGTAGCCGTTGCCAGCTCCAGTTTCTTAAGGCAGGCATCTGGTAGCGCGCATCACAGCAGGCCGGGCCAGGTCAATAAAAAATCGAGTCAGCCGTGGGCTAAAAAACGCGACTAAAAAAACAATGCAGAGCCGCGGTTAAAGACAGGTAGCCGAGCTAAGCGGTAGGGGAGAGGGGGATCAGGATCATTTGTATACTCGGGGAATGTGCCCGACCCGGTACACTCGATGCCAAAACGAACACGCCGACTTATACAGATGCCTATCTGACAATACGGACACTTTTAAAAAACACTTTTTCGGTTTTTAAAAGTTAAAGCCAACAAGCGCTGTGTTTTACACAATTCTTCCAATTTCCATTCCCAAGTTGCAAAAGTGAAACGTCCCAAAATATTTTGTCGCGCAAATCAAAAGTATATTTTATTCATGAGGAGCTCGTGTAATTTTTATGTAAATTTTAATTATTGATAACAAGGGACCATTGTTTGACGACACTTTCTTCGGCATGCGGAGCTCCTTGTTTTGT
Sequence-based reagent	Tc_klu_8p6	This paper		TTATGAGTTTGGTTATCGTCGGAATCGGGCATTCTTCCTTTGTATCCGATTTTTAGGTAACAAGCTAGAAATTCCAGAGCTACACCTACGATCCATCAAGTCGGAGCCGTTCTAATTGGCCGCTCCTATCTATCGTCTGAAGGAGGCAGCAAGCAGCACACGAACACCTGGCTGCCAATGCACCTGATGCGGTCGTCCGTCGCTGCTATCAACTCACAATGTTTACTTGTGCTTACGCCAAAATTATGACGAATATTAATGCGGCCTCGTACGCAGGCAGGCATGCGGCGTAATTACTACCGGAGGGACCTTATCTCGAATTATTATGCAGCAAGCAGCAGTGAAAAATAGCGCAACGCCTGCTGCACTCATCCAGATCTGACAAAGATGAAGACGTCGCTGACATCTTTATGATTGTGCTTTTATTGCACCTTTTCGCGGAATTCCGACCATTCGAAGCGACCTTTGCGCTACGGAGAAGAGGAAATTTACACCGGGAGTTGACTTATGATGGGAGAACCACTCTCAACGAACGCAACTACTTTCCAGGAATATGAGAAGAGTGCTTAACTGACAAGTCCAAATTCGAACTTGAGGTTA
Sequence-based reagent	Tc_ush_6p8	This paper		TAAATAATCCAGACATGCATTGCATGTAAGTATCAGAGATACACGGTAAGAGTGGAGCTTTTCGAGAATCCGGAAACCGATCAGATAAGTTCTGAAAATGACTCGTCCACGAATAGATTTAGGATCGGAGCTGTTTTCCATTCGCCGAGATAAGTCGATAAGTTTCAATAAGTCCGAGTTCTGGCAACAGCCAGCACGGTACGGGCTGCCGCCGTCTTGGTTTCCAGTTTTCTCCAATGTCGTGGTATTAATCAGGGCGTTATCTCTAGCACATAAACACACGTATGTGTATGTGGGGCGATGTCGGTGGCGATACCGTTCCATGTGGGGGTGTAGCTGTTGGGGGTATACGGGCCTGTTCGCCGTCCGATAGCGCGAAAGATACGACCTGGAAGTAGGAAACGAGACAGCGAGATAAGAAAGTAATATGGCGGCTGCTGCAAAGAGATAACGACTGATACGCGCCTGCACCTTTCCCGACCTGCAACTCTACGTGCCCATTATTTTTGGAAAATTCAATGAGAAATCCG
Sequence-based reagent	Am_ex_20p3	This paper		TCTTGAGATCTTTCTGCATATAGCCGTGGTCTTCTTGCCCTCCCTCGCCCTCTGGCCCCGGACACCATCCACGGAGCTCCTTCCCTTCCCTTCACCGAATATACTCGGCTGTGCAGCGCCTGCTACCCTCTCGCTCTACTCTCTTGCCTTTCCACCAGTCATGACAAGCCGGTCCGACTGGTACCCCCACCAACGCGGCCGGACGGACCCTTCTTGGGCCTCGCAAGGGCCCTGCGGGACCCCTCCCTCCTACATTCCAGCGGGGCCCCATCACGGCGAGGCTGAGCTGGCGGGTTTTGAGGCGCGCGAGCCATGCCACGACAGGAAAAAAATGCATCTGAAAAACGAAAACAAGTAGAAAAAGGTGGCTCACACCCCTGCATGCGTGCGTCGGTTTGCGTGAACGTTGCCCGGACCCCGTACCGAGGCCTCCTCCTCCTCCTCCTTTCTCCTCCTTTCTTCCTTCCTTTCTTTTCGCTCCTCTACCTCCTCTGCGCGCCTCTTTACGCTCCTCTTCTTGCTCTACGCTCTCGCGTACGTGCCCGCAAACTGCTGCCTGCCTGTTCAACGCTTCTTCTTCGTTTCCTTCTTCTTCTTCTTCTTCTTCCTCCTCTCTGCTTCGTCCTTGCGTTTCTCTCGATTCGCGTCACATCTCCGCTCCCCCAAATACGTTTCCTTTCTAAGATCGTTT
Sequence-based reagent	Am_klu_20p2	This paper		TTATTTATCGCCCTCGAAAGCGCTCGTCCTCTGCAGATTTCGATCGAGTCGTTCGACTTCGATATAAGAATTTCAGTGTAAACGCGATACACGTTAAATAACGAATATTTACGGACAAAGTCGGGCGAACGACGCGATCCTGGCCGCTCGTGGCCGATGCGCAGGAAGGTAGGAGAGCGGAGAGGTTTTACGCTGTTCGCGGAGAGGAGGATAGGTTCGTATAGCTCCTTAAATCAACCCTAGTTGGCCTGTCAACCGAGTTGGCGCGCGCGCGCATTCCCTTTCGCGGCGCACAAATTACCACGCGTTTAATTACCGTCCGATATACGAAGCAGGCTCATTAATCACCACGCCGATAACCCGTAATTTTCCAGCAACGATAAAATCTATCGCGCGACACCGGCTCTCGCGACTTTCCTCTCTCTCTCTCTCCCTCTCTCTCTCTCGTTCGAGGAGAAAGGAGAAAAGGAAGCAGGAGGACGGAGGAGGTGTGCAGAGCGATCCTGTCGCCGCTTCCATATAGATTTTTTTTCTCCCTTCCGCGCTCTTTCTCGCGCCAGTTCTCTTCGTGCGGCGGAAAATAGAGCGGCGCAACTCCCCTTCTCGCGACTCACGGAGGGCGAACAGCTGAAGCCGGCCGATCGATACGAA
Sequence-based reagent	Am_ush_20p8	This paper		GGGGCTCCCTCCTCCTTCCCTCCTCCTCCGTCTGTCCCAGTTGGTCAGCCACGGTATCGTTTCGACGTCGATTCATCCCTCTTTTTCTCCCCCCTCTTCTCTCTCTCCTTCTGCCCCTCCCCCTCTCCCCTCCTCGCCATCGGCTTCGAGAGCCACGAGGCGATCGAGAGAGAGAGAGAGAGAGAGAGAAAGGGTACCCCATCGATGGATCGATCTATCGATCCACACGGGGATCCACCACGCTCTTCTGCCCTCTCCTCCTCCTCGCCACAATTTCTCTCCTCTTTACGTACGCTTCTCTCGTCCTTCGTGCCGCTCTTCGTCGCCATCGAGATTACGGCGAGCGAGGGGCCGACAGCCGAGGGGCTTCTTCCAATACTTTGTAAGTTTATTTGTATGATCCGCCAATACTTTGTATCTTTATTTATATGAAATCGGATGGCGGATCGAGATTGCTCTCTCTCTCTCTCTCGTGTCGCTCGTGTCTCGTCTCGCTTCTCCCCCCGTTTCCTCTTAAAATTAATTATACGTCCAAGGTGGGCGTAAGAGAGAGAGAGAGAGAGAAAGAAGTCGCAATGAAACCGGAAGGATAAAGAGAATCCGATGGTGCGCACACGCACGTGTATGTACACGTCCACTTTATAACACTCG
Sequence-based reagent	Aa_hth_35p9	This paper		TTCCGAACACCTTGATTCAAATCCGAACAGTAGGTACGAATAAATCATACCGTTTTGCTTCGAAATCTGGACACCTAAGACGAAGTGTATTTTCAAACTTGAATATATATATAGTAGAGATGGTCGGGTTTCACATTTTTCAAACCCGAACCCGACCCGTACCCGACTTATTTTATTTCTTCGAACCCGGACCCGACCCGAACCCGAGACCATAATTGAAAAGCAAACCCGGACCCGACCCGAACCCGAAAATTTTTCACAGTGCAAACCCGAACCCGACCCGAAACCCGAAAAATGTTTGTAAAAAAACCCGAATACAACCCGAGTTTGAAAAGATGGTAAATTCATCGTTTCTGATGCATAAAGAAGCTTTTAGATTGTTACTCTGTTCACAATTTTCACCAAACCCGACCTGAACCCGATTCAAACCCGACTTTTTGTAAGCCCGAACCCGACCCGTACCCGATAATTTCGTAGCCTACAAACCCGACCCGAACCCGAACCCGAAAAATTTCAAATATTCAAACCCGAACCCGACCCGAACCCGTCGGGTTCGGGTTCGGGTCGGGTTTCGCGTTTGAAAACCCGAGACCCGACCATCTCTAATATATAGGCAAATTCATACATACTAAAAATCCAATGGTGATTCTTGCTTCGAAATCCGGACAGCATGTGAGAGCCGATTCAAATATTGGACACATTTGCTTCGAATTCCGGACACTTCTATTTATCTAGTTTGTTCAAAGATTC
Sequence-based reagent	Aa_ubx_26p0	This paper		AAGTCGGGTTTAGTCGGGTTTGTGTCGGGTTTGGTGAGAATTGTGAACAGAGTGACAAACTAAAACCTTCCCTATGTATCAGAAACGATGAATTTATCATCTTTTAGAACTCGGGCTTTATTCGGGTTTTCCTTAGAAAACTTTTTCGGGTTTCGGGTCGGGTTTGGGTTTCAACAGCGAAAATTTTTCGGGTTCGGGTCGGGTCCGGGTTTGATTTTCAATTATTGTCTCGGGTTCGGGTCGGGTCCGGGTTTGAAGAAATAAAATAAGTCGGGTACGGGTCGGGTTCGGGTTTGAAAAATGTGAAACCCGACCATCTCTACTCTTCAGGTAGTCGAGAGTTGTTTTTTTTTATCTTTTATTTTTATTTTAAAGGCACTCTGTGCTCGTGCCCACTACTATGCCGAAATCAGTTCATCTGTATCTTCTTCACCGATTAAGATCTATTTTTAACTAATCTATATTTAAATCTACTTTCACTCTCTTCTACTCGTTTGCTCTCATACCGAGCAGGTAGGAGAGTGCTCTGCTGATAGTCCAATCGATTTCCATAAGCCATAGTTCCATTGCTCTTGCGGTGGTTCGTTTTGCCATGTTCCTGAGTCGTTTGAGGCTAGCTGCCTGCGAAGTGGGTCAGTTTGTCTCAG
Sequence-based reagent	Aa_psq_21p5	This paper		CCGAATTTGTGAGAGAGATAGGAGCCAATGTTTGAGTGATTCCCGCGAAGAATTGAAACCTATAAACGATTCCCACTAATTTTTGCAACATCTGTGATTTTTGATTTGATTTGAAACTGCAACTGACAGAAGATAATCAAAATACACTTTTTTCGCATTCGTACATCAATTGACAACCATCACTTGACACACCTGGCGATATGGACCAATAGGTCTGTGCCACAATAAGGGAGAGAAAAAAAAAAAAAAAGTGTGAAGCAAAAACACGCACATGTAACTTAAAGCACCACAAGAACCCTTTCAGCACCGGCCGCTTATGCTGATTTTATTAAAAAGCTTTATGCATACATGTACATAAGAGTGAGCATGCCGAAGCTCGAAAGTGTGTGTATGTGCGAATGCGCCAAAACACGATTATGTTTTCGTTTGTATTTCTTTCTTTTGCCGGCAAAAATTCTGTGTTTCGTTTTTTGATAGTAGGTAACTATGCCCACACAGTTACGGATCACACATAGTCATGGATCACTTTGGCGTTCAACATCGGATAACTCGCTCAAAACATATTTGCATGTGATGTAAACATATTTTTGCCAAGTCATAATATTTGTCTTCTGTCATTTGTAATATCAAATAATAAGCATAACATTAAACCGCAAAATAATGGTGTTTTTGAAAAATGTTTAGTTTGTATTGCCAAGCTATCATTAAATAGTCATTTATTGTAAGAAGTGCCATCAGCATTCTCCTATGCTTTTAGGTGATAAAATTCAAATATTATACATAATAGTTCCTCGTTCTCTTGAATTCAGTATGATTTCTTTGTTAGAAAACATTTTTCTTGTTTGTTGATACTGAATATTAGCAATTCCAACTAGTGATATTAGCAATTC
Sequence-based reagent	Tc_hth_15p5	This paper		TAATCTTTTAATTTAAAGCGTAGCTGAGCAGCTGGCTCTAATTCCACTTTCCTTATTTGGTTTCGTTGGTGTGGATTTTTGAAACGGATTATTTCGAGAAATAATAGTTTATTAGTGGTGGAAATAATGAATGGGTCTGGAGCGAGTTCCAGAGTGCGATTGGTTGGTTAGCGGGTAAATTTTTAAAAAGTGGGTGTCTTCTCCGACGGCAATTTAACGATCGTAACGACGTCGTCGCTAATTAGGCTCGTTGAGGCCGTCGCTAGATCGATAACACAGGCTGCGACATCGTCACAATGCACCGGTCGGGTTACACATCGGAGTCCGTCTCCCGGGGGCCCGTCTCAGATTCTCCGTATTAAAACACCGACATGTAAAAATATGGAAATTGCGCGCGGCAGAATGCGGTCCGATCAACCGGATGGCCATCGCGCATCGCTTTGCATTCGCAGCCGCATTTAAATTGCTAAAAGGGGACACTATCGAGCGGTCCATCTCTCTCGCAGCGTTGCGATATTATAATCTTGTTGCAAGGTAAATGCACATAACCGGTTACCCCAGACAGACGACGTCTTTGACACGAAAAAACCTGCCATCTATGTACAGCGGATCCTAATTTACGGCCTTATTCCATGTCATTAAGAGCATACGGGACGGACACGTTTTTAGGAACTTCGGACCCGACTTATCTCCGCGGACCGATAAGGAAATGTGCCTCTGGACACCTAACTTTGCCGACCAACAAAATCATAACGCTCGCTCTATGCCCATTGGGCAACACGAAAAAACCTGCC
Sequence-based reagent	Tc_Ubx_17p4	This paper		TGCATGTATGTCGAGTGGGTCCGGATGATGCGAACTCCCGCCGATTTCTTCGCAATCTGCAAATTCGCTCAAGTAGCTTAATAACAATGACAAAAGTGAGGCGGTATATTTCCGGCCGTCCGTTGAAAATTGTAATGATGTTATTAAAATTATGACGTGGCCGTGATGGTCGCCGAATTCTGGCGAAACGGCCGCGTAAAAACGGCACATAATTGGCTGACATTAAGATGTATCTGGAGATGTTTTTCGAATGCCTTCGTCCGGCGCGAATGCCTGAATAAGCGGCAAAGCTCGGAAAGCTCTTATAAATAAAAATGTACGGAGCCAATCAGATCGGCGAGTAAAAAGTACGTCTTTTCTTACACCAGAGGATCGCAGCTGCCGCAGAATCCGGTCGCGGATAAGAAATAAGAAGTGCTGCATAAATGCATTGATCATTCGCCGGGTCTCCGTCTGCTGTTCCTCAGCGAGAAAACGGGTTTAAGTCTGGATACTTTTGGCTCTCTGGAAAGTGCTTTTTGCATTAAGCTGCCGAGAGAGAATAAAGACGTTTGCGGTGTCGGACGGTGACCAATGCTGCTGCTGCTGCTCTGCCTTCCAAGTGCGTGCTTTAAATCTTCCACTTTGCAAGTAAATCGAGACGAACGCTGAATATTTTACACGAACACTGTTTATAGCCCAAATAACAGCCTTCCAAGGGCGGCCACCATCAAAAAATGGAGCGCTCAAACCCGAAATATGGGCGGGCGAAAATTATTCAAACCACAAAGCGAGGAAATCAGAAATTCAAAAATTGACGGCTTTCAACTCAGGACTGAATTTTTATAAATTTTTGTTCGCTACTGCAATTTGGGACAGAAAATTACATCT
Sequence-based reagent	Tc_Ubx_19p9	This paper		CGTATTTAAATATCGTTAGGTTCGATGGTAAAATTGGAGAAAATTGTCGCGCGCGTTTAAGACAAAGAAAATTCCCGTCGGGTTATCAATCTTGGGTTATCTGTACCCTCGG GCCGAAAAACTCTGTAAAGAAGAGACAAAAGGACGTGACAGTCCAATTTCCATTTCAGATCGAAATTGTTCGCCCCCCGGAAGTTTATCGGGGCCCGTTGGCGGAATTAATAAATTGGTGCGCGACTTAATTGCGGCGATAAAGAAGAGAAGAACACGAATGAGGGACGGCGACAAAAATATTATTTGCTCGTGAACGAGGAGGCAAAGGGCATTGATATCTCGTGCAACGCCGGATATTGGCTGCTTCTGGTCGCGGTTTGCGGGGCTTCTAAGACTGTGCAGGGTTTGGGGAGCGGCCCCGAGCTCGAGAGAAATTATGTACGAGGCATTGGGAGCAATATATCTCCGGCGGGACGTGCCAGACAGAGTAGACGGGGTATTATATAGGAAGGAAGGAACCTGAGGCCGGGGCCGGAGCCTCCTCGTCCCCAGGCGCTCGTCCCCCAGAATGAGACACTTGCCGCCAAGTCCACCGCCTTAAATTGTCATCTGAAGAAAGAAACTTCATTACGAACTACGCCCTCATTTCTTTGCGAGGCGATCCATCGCGCAAAAGCAACGCACGCATTTTGCAACAACTATTCAACCACTAAAATTAAACGAATTTCAAACCTATTCCGGATTAATGATTTCCTCCTCGATTCAAGCTAATTGGGTGTTTCCTAG
Sequence-based reagent	Tc_psq_19p7	This paper		GGATTTTTTAGATAGATCATCAAGTTAAAAGTGCTTCGAATATATGTCATCAAAAATAAGATCAACTGATGGCTTTCTTTGCTTTATTCCCAATCTACTGTTAGAAAATCAACAACAACTAAGTTTTCTGTAAAATATAGTTCTTTCGGTGGCAAGAATAATATTATAATCGGGTTTCTTCTGCCTTATATTCTGTTTTCTTTGCTCCTATGTTAGTGCAAGTGTGTAACTTGGCGAACTCTTTCGAATTATCAAGGAAGTGTGAGTTTTATGAGAAAACAGCTAAAGTCGCCCCTAATTTGTTGACTTATTTGCTTTCGTTGGTTCTCCCGTTCTTTGGAGTATGTCGTCCGGTTTTTCTTATGAGCCATAATTACAAATTTCCATTTTCGGTTTTCGGCTCGCGTTCGTTTTGGAAAAGAGCGAATGTGCGGCGCGTTCATTTTCAATTTTGCGCGACCGTCCGACATTTTCCAATTTTCCGTGCAAGGACGAGGAGCGAGTGCAAAAAATGGCAGTCCTTGTCTGCAAAAAGCCCCAATTAAAACCGAAGTTGTAGTAGTGCGTGCGCCGAGCATTTCTCTCGATCTATCACGGGGTAGCAGCATCCCTCCGTAGGCTCACTCTCTGGCCAGTCTTAGTTTGCGCTTTCCCCGGAATTCACTGAAGGTCGTCGAGGTCGCAAGTAAGTACACAGTGCATGTGCACTTGCATGCATGCTTGCACTTTCTGTGCCCCCGCGCGCCGCCGCCGCCGCCGCATTAGCGTCTCTGTTTTGGTCCTTATATCCATCCGCTGTTCCCTTCTTCTGTCTATCCTTCAACTTCCTTCGCCGCTCGCCAGCTCCGGGACGCCACTCCATCATAAAACTGCGACCGCAAAAGCGACACTCATTATCGATTGCTCCAAGACGAATTAAAAGCCGCAGCGCTCCCCAAAACCGGGTTATTTTTTTCGGAATTTTGCTGGCTTGGAGCGGACTCCCAGACGATCCCCGGACTAATCCGGAGGGTTGCCTGGCGAGCGGCATTCGGCTTTAGGCTCCGGGGCACGCATTGGGGGAAAGTGATGCGGTGTCTGGACCAATCAATACCGGGTTCAAGGACGGCTTCTCTTATATGTGTATGTGAGCTTCCTTTTCCCGCTCGTCAAAACGGGACAAGACGGGAATTAATTGCACGACAATTGGGACGCCGACTCCACAGATGGGGCGACAAAATGGACGCAACGAACTAAATCTATTCACTTTT
Sequence-based reagent	Am_Ubx_0p39	This paper		TCTCTCTCTCCTCGAGTGTAGCATATATCCATTCCACCATCGATCGAGGATTTCGATCCCCCTTGGACTCATGCTGCGATATTCGATCGTCCCTCCCCCCACTCCTCCGCGCTCTCATTCGATCCTTCTTTTCTTCCCTCCCCCCCAACCACTTTGATCCTTCTCTCTCTCTCTCTTCCTTCTTTTTACTTCTTCTTCTTCTTGCTGCTGCAACTACCCGCTGCCTCTAACCGCTAGCCGGACAAAACATTTCTTAATTGGGTTTCGTTCGGAAAGAACCGTCCGATTTCGTTTCGCAAGGGATCCAGCCTGCTGCTTCTGCCGGTTTTACCGCGTCTCTACGTGGCTTCTGTCGTTCCCTCCTCGTTCTGCTTGCCTTTCCTTCGAACGATTATTTATTTCGTCGTTCGAATTCCTTATTTTTCCATCCTGTTATCCCTTATTGTAATAAAGTAAAAATAATTGAATTTTCCTTCGAACGAGCGAAGTTTGTTCTAATC
Sequence-based reagent	Am_Ubx_37p2	This paper		AGAGAGAGAGAGAGAGAGAAAGTCAGGCAGACGGAGACAGAGGAATGGGTTGGGTAAGGGGGATAGAGTAGGGGCGGGAGGGCGTTCCACGGCACCCTGCATGGGGTAGCTTGCAACCTCACGCGACACTAGAGCCATCTATATCCCCGGAGATTTATGAGTTCCTGGTGCAGCGGCTGCTCGCAGCAACTACACACCACGCAGTATCGGGTCCGGTGTTGGTGCTGCCCCCTGTCGCGACGGGCGTGCTGTTGCCCCGCGGGGGTTACGCGCAATTCGCGCTCCGTGCAACGTCGCCCTGATAAAAAACTCTTGCGACTCGATCTCAATCCCGATGCTTCTGCGAACCTTCCCTCCGTTCTCGCGCCGTCTCGTCGTGCGCCCGTCGCGGTCGTCTCTCTCTCTCTCTCGCTCTCCGGTGTTGGCGGGCTATCGGATCTTCTCTCTCTCTCTCGCTCACTTGGTTCGCTTCTTGCTTTCGATGCGACGCGACGACCAGCGATCTCACTTTCTCTCTCGCTCTCGCTTTCGAGCTCGCACTTGAAATATCGATCATCATTGTGTTTCCTACGCATTTGTAGACCGCAAACGCGAAATTATTATGGGCCTGTGCACGTTTGAATTTCTTATATTCTTTTTTTTCCATTATACGCTAGGTTAGCGTAGATATAATTCTGCTAAATATAGTGAAGATAATTCGAATTTAAATTAAATTGAAAATTTTCGTATTACATAATACTGTTTCGTTATTTATAACTGATTAGAATATTTATTGATACCAAATGAAATTTTTGGTAAACTCTCGAACATTGTTTCATTCTTCTATATCGTATTGGTGAAAAATTACATCTCGATTTTTTCTCACGAACTTATATCGCGGTAAAAGAACTGTGGACAACTGTGCAGCATCTCCTCGCTCGATGAAGTCATTTGAACGAGCATTCCTCGGCCGATCTCAGATACAATCTCCTTCAAACAAAGAGCTCCATTGCCGCGTGCA
Sequence-based reagent	Am_psq_29p2	This paper		TCGCACGAGTACATAACGCTACCTTTGTCGCGTCGAAGGTAGAGGCACGATTCTGTCCTTTCCCGTTCTCTCGCGAACCTTGCATCCGTCTTCGTCTCGCTGTGGCCAAACGCGTGCTAGGTCTTCGTCTTCCACATTCCGTCTCGTTCGTTTCCGCACAGACTATATTTCTGTTCTCGTTTAGCCGCGGAAAGTCTTGCTCGCTCCCACGGGAACCACTCGTCGATGCTCGTCGCTTAACCGTCAGAGGCGAGCGCGCATTTCTCTCAAACACCGCAGACTTGCCTCTCCGCCGATCCCCGTTCCCACCCCCGGTGCTCGATGCTCTCTGTCACCCCTCCACCAAACGGACTCCTACCGGCCCGCTCCCCTCGCTTTGCGCCGCTTTCCACCAACCGTCCTGCCACCCGCCGGTTTTCAACCCCTTTCCCCGCTCTCTCGGCGACTGGTCAGGTGCGCTCGCTCGCTCGCTCGCTCCACGCGTACGCTCAATCGCTCTCTGTCCACCGCCGAGCACGCATCCCCCGCGAGTCTCTTCCTCGTTGTACGCGCTCGAGCGCGGATTCAATCCGTCCTTGTTCGTCGCGTCGGCGAATTTCGCGGCGTCCTCCGCCGCCGCCGCCGCCGCCGCCACCTCTTCCTCCTCCTCCTCCGCCTCCTCCTCCTGCTGATACTCCTCTTCCTCCTCGGT
Software, algorithm	SCRMshaw pipeline	Asma et al., 2024, doi: 10.17504/protocols.io.e6nvw1129lmk/v2		
Other	REDfly database	Keränen et al., 2022, PMID:35886794	RRID:SCR_006790SCR_006790	Database with results information, see “Results—An insect regulatory annotation resource”

## References

[bib1] Aldaz S, Morata G, Azpiazu N (2005). Patterning function of homothorax/extradenticle in the thorax of *Drosophila*. Development.

[bib2] Antosova B, Smolikova J, Klimova L, Lachova J, Bendova M, Kozmikova I (2016). The gene regulatory network of lens induction is wired through meis-dependent shadow enhancers of Pax6. PLOS Genetics.

[bib3] Asma H, Halfon MS (2019). Computational enhancer prediction: evaluation and improvements. BMC Bioinformatics.

[bib4] Asma H, Halfon MS (2021). Annotating the insect regulatory genome. Insects.

[bib5] Asma H, Halfon M (2023). Software Heritage.

[bib6] Asma H, Halfon M (2024). Software Heritage.

[bib7] Asma H, Liu L, Halfon MS (2024). Protocolsio.

[bib8] Barth NKH, Li L, Taher L (2020). Independent transposon exaptation is a widespread mechanism of redundant enhancer evolution in the mammalian genome. Genome Biology and Evolution.

[bib9] Benson G (1999). Tandem repeats finder: a program to analyze DNA sequences. Nucleic Acids Research.

[bib10] Benton ML, Talipineni SC, Kostka D, Capra JA (2019). Genome-wide enhancer annotations differ significantly in genomic distribution, evolution, and function. BMC Genomics.

[bib11] Boedigheimer M, Laughon A (1993). Expanded: a gene involved in the control of cell proliferation in imaginal discs. Development.

[bib12] Boyle AP, Davis S, Shulha HP, Meltzer P, Margulies EH, Weng Z, Furey TS, Crawford GE (2008). High-resolution mapping and characterization of open chromatin across the genome. Cell.

[bib13] Bozek M, Cortini R, Storti AE, Unnerstall U, Gaul U, Gompel N (2019). ATAC-seq reveals regional differences in enhancer accessibility during the establishment of spatial coordinates in the *Drosophila* blastoderm. Genome Research.

[bib14] Buchberger E, Bilen A, Ayaz S, Salamanca D, Matas de Las Heras C, Niksic A, Almudi I, Torres-Oliva M, Casares F, Posnien N (2021). Variation in pleiotropic hub gene expression is associated with interspecific differences in head shape and eye size in *Drosophila*. Molecular Biology and Evolution.

[bib15] Buenrostro JD, Giresi PG, Zaba LC, Chang HY, Greenleaf WJ (2013). Transposition of native chromatin for fast and sensitive epigenomic profiling of open chromatin, DNA-binding proteins and nucleosome position. Nature Methods.

[bib16] Cannavò E, Khoueiry P, Garfield DA, Geeleher P, Zichner T, Gustafson EH, Ciglar L, Korbel JO, Furlong EEM (2016). Shadow enhancers are pervasive features of developmental regulatory networks. Current Biology.

[bib17] Carroll SB, Grenier JK, Weatherbee SD (2005). From DNA to Diversity. Molecular Genetics and the Evolution of Animal Design.

[bib18] Carroll SB (2008). Evo-devo and an expanding evolutionary synthesis: a genetic theory of morphological evolution. Cell.

[bib19] Chua EHZ, Yasar S, Harmston N (2022). The importance of considering regulatory domains in genome-wide analyses - the nearest gene is often wrong!. Biology Open.

[bib20] Claringbould A, Zaugg JB (2021). Enhancers in disease: molecular basis and emerging treatment strategies. Trends in Molecular Medicine.

[bib21] Cohen SM, Bate M, Martinez Arias A (1993). The Development of Drosophila Melanogaster.

[bib22] Crosby MA, Gramates LS, Dos Santos G, Matthews BB, St Pierre SE, Zhou P, Schroeder AJ, Falls K, Emmert DB, Russo SM, Gelbart WM, FlyBase Consortium (2015). Gene model annotations for *Drosophila melanogaster*: the rule-benders. G3: Genes, Genomes, Genetics.

[bib23] Cubadda Y, Heitzler P, Ray RP, Bourouis M, Ramain P, Gelbart W, Simpson P, Haenlin M (1997). u-shaped encodes a zinc finger protein that regulates the proneural genes achaete and scute during the formation of bristles in *Drosophila*. Genes & Development.

[bib24] Deem KD, Halfon MS, Tomoyasu Y (2024). A new suite of reporter vectors and A novel landing site survey system to study cis-regulatory elements in diverse insect species. Scientific Reports.

[bib25] Dönitz J, Gerischer L, Hahnke S, Pfeiffer S, Bucher G (2018). Expanded and updated data and a query pipeline for iBeetle-Base. Nucleic Acids Research.

[bib26] Evans CJ, Olson JM, Ngo KT, Kim E, Lee NE, Kuoy E, Patananan AN, Sitz D, Tran P, Do M-T, Yackle K, Cespedes A, Hartenstein V, Call GB, Banerjee U (2009). G-TRACE: rapid Gal4-based cell lineage analysis in *Drosophila*. Nature Methods.

[bib27] Fishilevich S, Nudel R, Rappaport N, Hadar R, Plaschkes I, Iny Stein T, Rosen N, Kohn A, Twik M, Safran M, Lancet D, Cohen D (2017). GeneHancer: genome-wide integration of enhancers and target genes in GeneCards. Database.

[bib28] Frankel N, Davis GK, Vargas D, Wang S, Payre F, Stern DL (2010). Phenotypic robustness conferred by apparently redundant transcriptional enhancers. Nature.

[bib29] Giresi PG, Kim J, McDaniell RM, Iyer VR, Lieb JD (2007). FAIRE (Formaldehyde-Assisted Isolation of Regulatory Elements) isolates active regulatory elements from human chromatin. Genome Research.

[bib30] Gramates LS, Agapite J, Attrill H, Calvi BR, Crosby MA, Dos Santos G, Goodman JL, Goutte-Gattat D, Jenkins VK, Kaufman T, Larkin A, Matthews BB, Millburn G, Strelets VB, the FlyBase Consortium (2022). FlyBase: a guided tour of highlighted features. Genetics.

[bib31] Grosveld F, van Staalduinen J, Stadhouders R (2021). Transcriptional regulation by (Super)enhancers: from discovery to mechanisms. Annual Review of Genomics and Human Genetics.

[bib32] Gschwind AR, Mualim KS, Karbalayghareh A, Sheth MU, Dey KK, Jagoda E, Nurtdinov RN, Xi W, Tan AS, Jones H, Ma XR, Yao D, Nasser J, Avsec Ž, James BT, Shamim MS, Durand NC, Rao SSP, Mahajan R, Doughty BR, Andreeva K, Ulirsch JC, Fan K, Perez EM, Nguyen TC, Kelley DR, Finucane HK, Moore JE, Weng Z, Kellis M, Bassik MC, Price AL, Beer MA, Guigó R, Stamatoyannopoulos JA, Lieberman Aiden E, Greenleaf WJ, Leslie CS, Steinmetz LM, Kundaje A, Engreitz JM (2023). An encyclopedia of enhancer-gene regulatory interactions in the human genome. bioRxiv.

[bib33] Hafez D, Karabacak A, Krueger S, Hwang Y-C, Wang L-S, Zinzen RP, Ohler U (2017). McEnhancer: predicting gene expression via semi-supervised assignment of enhancers to target genes. Genome Biology.

[bib34] Halfon MS (2019). Studying transcriptional enhancers: the founder fallacy, validation creep, and other biases. Trends in Genetics.

[bib35] Halfon MS (2020). Silencers, enhancers, and the multifunctional regulatory genome. Trends in Genetics.

[bib36] Huang DH, Chang YL, Yang CC, Pan IC, King B (2002). pipsqueak encodes a factor essential for sequence-specific targeting of a polycomb group protein complex. Molecular and Cellular Biology.

[bib37] IUCN (2022). The IUCN list of threatened species 2022. https://www.iucnredlist.org.

[bib38] Jacobs J, Atkins M, Davie K, Imrichova H, Romanelli L, Christiaens V, Hulselmans G, Potier D, Wouters J, Taskiran II, Paciello G, González-Blas CB, Koldere D, Aibar S, Halder G, Aerts S (2018). The transcription factor Grainy head primes epithelial enhancers for spatiotemporal activation by displacing nucleosomes. Nature Genetics.

[bib39] Jory A, Estella C, Giorgianni MW, Slattery M, Laverty TR, Rubin GM, Mann RS (2012). A survey of 6,300 genomic fragments for cis-regulatory activity in the imaginal discs of *Drosophila melanogaster*. Cell Reports.

[bib40] Kantorovitz MR, Kazemian M, Kinston S, Miranda-Saavedra D, Zhu Q, Robinson GE, Göttgens B, Halfon MS, Sinha S (2009). Motif-blind, genome-wide discovery of cis-regulatory modules in *Drosophila* and mouse. Developmental Cell.

[bib41] Katzen F (2007). Gateway recombinational cloning: a biological operating system. Expert Opinion on Drug Discovery.

[bib42] Kazemian M, Zhu Q, Halfon MS, Sinha S (2011). Improved accuracy of supervised CRM discovery with interpolated Markov models and cross-species comparison. Nucleic Acids Research.

[bib43] Kazemian M, Suryamohan K, Chen J-Y, Zhang Y, Samee MAH, Halfon MS, Sinha S (2014). Evidence for deep regulatory similarities in early developmental programs across highly diverged insects. Genome Biology and Evolution.

[bib44] Kazemian M, Halfon MS (2019). CRM discovery beyond model insects. Methods in Molecular Biology.

[bib45] Keränen SVE, Villahoz-Baleta A, Bruno AE, Halfon MS (2022). REDfly: An Integrated Knowledgebase for Insect Regulatory Genomics. Insects.

[bib46] Klein T, Campos-Ortega JA (1997). klumpfuss, a *Drosophila* gene encoding a member of the EGR family of transcription factors, is involved in bristle and leg development. Development.

[bib47] Kuznetsov D, Tegenfeldt F, Manni M, Seppey M, Berkeley M, Kriventseva EV, Zdobnov EM (2023). OrthoDB v11: annotation of orthologs in the widest sampling of organismal diversity. Nucleic Acids Research.

[bib48] Kvon EZ, Waymack R, Gad M, Wunderlich Z (2021). Enhancer redundancy in development and disease. Nature Reviews. Genetics.

[bib49] Lai YT, Deem KD, Borràs-Castells F, Sambrani N, Rudolf H, Suryamohan K, El-Sherif E, Halfon MS, McKay DJ, Tomoyasu Y (2018). Enhancer identification and activity evaluation in the red flour beetle, *Tribolium castaneum*. Development.

[bib50] Laiker I, Frankel N (2022). Pleiotropic enhancers are ubiquitous regulatory elements in the human genome. Genome Biology and Evolution.

[bib51] Lewis EB (1978). A gene complex controlling segmentation in *Drosophila*. Nature.

[bib52] Li L, Zhu Q, He X, Sinha S, Halfon MS (2007). Large-scale analysis of transcriptional cis-regulatory modules reveals both common features and distinct subclasses. Genome Biology.

[bib53] Lindhorst D, Halfon MS (2023). Reporter gene assays and chromatin-level assays define substantially non-overlapping sets of enhancer sequences. BMC Genomics.

[bib54] Matthews BB, Dos Santos G, Crosby MA, Emmert DB, St Pierre SE, Gramates LS, Zhou P, Schroeder AJ, Falls K, Strelets V, Russo SM, Gelbart WM, FlyBase Consortium (2015). Gene Model Annotations for *Drosophila melanogaster*: impact of high-throughput data. G3: Genes, Genomes, Genetics.

[bib55] Mazo-Vargas A, Langmüller AM, Wilder A, van der Burg KRL, Lewis JJ, Messer PW, Zhang L, Martin A, Reed RD (2022). Deep cis-regulatory homology of the butterfly wing pattern ground plan. Science.

[bib56] McKay DJ, Lieb JD (2013). A common set of DNA regulatory elements shapes *Drosophila* appendages. Developmental Cell.

[bib57] NCBI (2024). Genome.

[bib58] Osterwalder M, Barozzi I, Tissières V, Fukuda-Yuzawa Y, Mannion BJ, Afzal SY, Lee EA, Zhu Y, Plajzer-Frick I, Pickle CS, Kato M, Garvin TH, Pham QT, Harrington AN, Akiyama JA, Afzal V, Lopez-Rios J, Dickel DE, Visel A, Pennacchio LA (2018). Enhancer redundancy provides phenotypic robustness in mammalian development. Nature.

[bib59] Perry MW, Boettiger AN, Bothma JP, Levine M (2010). Shadow enhancers foster robustness of *Drosophila* gastrulation. Current Biology.

[bib60] Perry MW, Boettiger AN, Levine M (2011). Multiple enhancers ensure precision of gap gene-expression patterns in the *Drosophila* embryo. PNAS.

[bib61] Prasad N, Tarikere S, Khanale D, Habib F, Shashidhara LS (2016). A comparative genomic analysis of targets of Hox protein Ultrabithorax amongst distant insect species. Scientific Reports.

[bib62] Qin T, Lee C, Li S, Cavalcante RG, Orchard P, Yao H, Zhang H, Wang S, Patil S, Boyle AP, Sartor MA (2022). Comprehensive enhancer-target gene assignments improve gene set level interpretation of genome-wide regulatory data. Genome Biology.

[bib63] Quinlan AR, Hall IM (2010). BEDTools: a flexible suite of utilities for comparing genomic features. Bioinformatics.

[bib64] Rickels R, Shilatifard A (2018). Enhancer logic and mechanics in development and disease. Trends in Cell Biology.

[bib65] Royal Entomological Society (2023). Understanding Insects: Facts and figures St. Albans, UK2023. https://www.royensoc.co.uk/understanding-insects/facts-and-figures.

[bib66] Ruiz JL, Ranford-Cartwright LC, Gómez-Díaz E (2021). The regulatory genome of the malaria vector *Anopheles gambiae*: integrating chromatin accessibility and gene expression. NAR Genomics and Bioinformatics.

[bib67] Sabarís G, Laiker I, Preger-Ben Noon E, Frankel N (2019). Actors with multiple roles: pleiotropic enhancers and the paradigm of enhancer modularity. Trends in Genetics.

[bib68] Sagai T, Amano T, Maeno A, Kiyonari H, Seo H, Cho S-W, Shiroishi T (2017). SHH signaling directed by two oral epithelium-specific enhancers controls tooth and oral development. Scientific Reports.

[bib69] Sanyal A, Lajoie BR, Jain G, Dekker J (2012). The long-range interaction landscape of gene promoters. Nature.

[bib70] Schember I, Halfon MS (2021). Identification of new Anopheles gambiae transcriptional enhancers using a cross-species prediction approach. Insect Molecular Biology.

[bib71] Segert JA, Gisselbrecht SS, Bulyk ML (2021). Transcriptional Silencers: driving gene expression with the brakes on. Trends in Genetics.

[bib72] Simon J, Peifer M, Bender W, O’Connor M (1990). Regulatory elements of the bithorax complex that control expression along the anterior-posterior axis. The EMBO Journal.

[bib73] Smith E, Shilatifard A (2014). Enhancer biology and enhanceropathies. Nature Structural & Molecular Biology.

[bib74] Suryamohan K, Halfon MS (2015). Identifying transcriptional cis-regulatory modules in animal genomes. Wiley Interdisciplinary Reviews. Developmental Biology.

[bib75] Suryamohan K, Hanson C, Andrews E, Sinha S, Scheel MD, Halfon MS (2016). Redeployment of a conserved gene regulatory network during Aedes aegypti development. Developmental Biology.

[bib76] Svácha P (1992). What are and what are not imaginal discs: reevaluation of some basic concepts (Insecta, Holometabola). Developmental Biology.

[bib77] Tomoyasu Y, Ueno N, Nakamura M (2000). The decapentaplegic morphogen gradient regulates the notal wingless expression through induction of pannier and u-shaped in *Drosophila*. Mechanisms of Development.

[bib78] Wang LH, Baker NE (2015). Salvador-Warts-Hippo pathway in a developmental checkpoint monitoring helix-loop-helix proteins. Developmental Cell.

[bib79] Wang LH, Baker NE (2018). Spatial regulation of expanded transcription in the *Drosophila* wing imaginal disc. PLOS ONE.

[bib80] Waymack R, Fletcher A, Enciso G, Wunderlich Z (2020). Shadow enhancers can suppress input transcription factor noise through distinct regulatory logic. eLife.

[bib81] Weinstein ML, Jaenke CM, Asma H, Spangler M, Kohnen KA, Konys CC, Williams ME, Williams AV, Rebeiz M, Halfon MS, Williams TM (2023). A novel role for trithorax in the gene regulatory network for A rapidly evolving fruit fly pigmentation trait. PLOS Genetics.

[bib82] Weisman CM, Murray AW, Eddy SR (2022). Mixing genome annotation methods in a comparative analysis inflates the apparent number of lineage-specific genes. Current Biology.

[bib83] Whalen S, Truty RM, Pollard KS (2016). Enhancer-promoter interactions are encoded by complex genomic signatures on looping chromatin. Nature Genetics.

